# Improving Reliability of Machine Learning Interatomic Potentials with Physics-Informed Pretraining

**DOI:** 10.1021/acs.jcim.6c00826

**Published:** 2026-08-28

**Authors:** Qianyu Zheng, Victor Fung

**Affiliations:** † College of Computing, 1372 Georgia Institute of Technology, Atlanta, Georgia 30332, United States; ‡ School of Computational Science and Engineering, Georgia Institute of Technology, Atlanta, Georgia 30332, United States

## Abstract

Machine Learning interatomic potentials (MLIPs) have emerged as powerful tools for molecular dynamics (MD) simulations with their competitive accuracy and computational efficiency. However, MLIPs often exhibit unphysical behavior when encountering configurations that deviate significantly from their training data distribution, leading to simulation instabilities and unreliable dynamics. This limits their reliability for materials simulations. We therefore present a physics-informed pretraining strategy that leverages simple empirical potentials to improve the robustness and stability of MLIPs for MD simulations. We demonstrate this approach through a pretraining-finetuning pipeline where MLIPs are initially pretrained on data labeled with embedded atom model (EAM) potentials and subsequently finetuned on the quantum mechanical ground truth data. Evaluation across three material systems (phosphorus, silica, and a subset of Materials Project) and three representative MLIP architectures (CGCNN, M3GNet, and TorchMD-NET) demonstrates that this physics-informed pretraining consistently improves both prediction accuracy as well as stability in MD compared to the baseline models.

## Graphical abstract

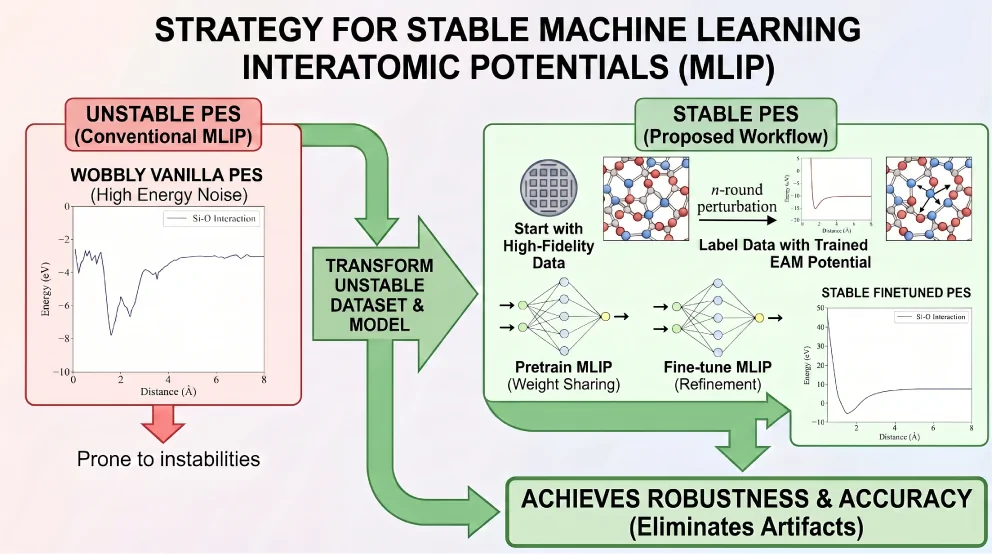

## 1 Introduction

Molecular dynamics (MD) simulations are powerful methods in computational physical science that capture the evolution of atomic configurations over time and enable investigation of diverse molecular, biological, and material behaviors and properties.^1–9^ A central challenge, however, is extending the spatial and temporal limits of these simulations while maintaining accuracy. This requires models to evaluate potential energy surfaces with both high efficiency and fidelity. Ab initio methods such as density functional theory (DFT)^10,11^ provide highly accurate evaluations of the potential energy surface with no parametrization required. While they remain the gold standard, their demanding computational scaling with system size severely limits their practical applicability for large systems or long-time scale MD simulations. This limitation motivates the search for alternative strategies that retain near-ab initio accuracy while reducing computational cost. Machine learning interatomic potentials (MLIPs) have emerged as a promising solution. By learning latent representations of atoms in various chemical environments without explicit knowledge of chemical bonds or interactions, MLIPs deliver competitive accuracy and computational efficiency superior to ab initio methods in atomistic modeling.^12^ This makes them particularly valuable for large-scale MD simulations.

Recent advances in deep learning have made end-to-end neural networks the dominant architecture in MLIPs. Early neural network potentials such as the Behler-Parrinello Neural Network (BP-NN)^13^ and ANAKIN-ME (ANI)^14^ demonstrated the capacity to learn potential energy surfaces directly from large data sets, though they still required hand-crafted structure representations. These were followed by graph neural network (GNN) architectures that represent atomic systems as graphs, with notable examples including SchNet,^15^ DimeNet,^16^ M3GNet,^17^ CHGNet,^18^ and ALIGNN-FF.^19^ To better capture physical laws and improve data efficiency, subsequent approaches incorporated E(3)-equivariance into their architectures. Equivariant models such as NequIP,^20^ Allegro,^21^ SevenNet,^22^ and EquiformerV2 ^23^ use irreducible representations and equivariant convolutions to preserve rotational and translational symmetries. More recent developments have demonstrated the potential of universal interatomic potentials trained on diverse, large-scale data sets, including MACE-MP-0,^24^ Mattersim,^25^ and Orb^26^ for inorganic crystals; MACE-OFF23 ^27^ for organic molecules; and AIMNet2 ^28^ for charged organic systems.

However, despite their impressive performance in predicting potential energy surfaces of complex atomistic systems, as evidenced by recent benchmarks,^29^ MLIPs exhibit concerning unphysical behaviors when encountering structural configurations that deviate significantly from their training data distribution.^30,31^ This challenge is particularly acute during MD simulations, which naturally explore configurational space through thermal fluctuations, structural deformations, and chemical reactions, and inevitably encounter regions far from equilibrium that are inadequately represented in training data sets.^32–34^ As recent studies note, aligning training data distribution to all configurational spaces explored during test-time MD may be intractable,^33^ and even foundation models trained on large, diverse data sets exhibit systematic softening that indicates incomplete coverage of the relevant PES.^34^ We use the term “out-of-distribution” (OOD) throughout this work in this specific sense to refer to MD-induced configurational drift relative to the training distribution rather than the broader supervised-learning notion of distribution shift,^35^ which is more directly addressable through data curation. In MD simulations, the consequences of this type of OOD error can be particularly severe: unphysical predictions propagate and compound over time,^36^ ultimately leading to unrecoverable trajectory errors and unreliable dynamics.^31^

Three main strategies have emerged to address MLIP robustness: active learning, physical constraints, and pretraining on large data sets. Active learning uses intelligent data selection to continuously expand the training data set and mitigate the OOD problem. Uncertainty-driven approaches by Kulichenko et al.^37^ and Zhang et al.^38^ guide data acquisition, while Van der Oord et al.^39^ employ Hyperactive Learning to automate integration of new structures into training data sets. Matsumura et al.^40^ focus on targeting unstable structures. However, active learning suffers from prohibitive computational costs due to iterative retraining cycles and expensive ab initio calculations. To address this, Sanjeev et al.^41^ propose sampling failing trajectories and retraining on system observables such as the radial distribution function (RDF) rather than labels from first-principles methods.

Integrating physical constraints into neural networks offers an alternative pathway to enforce physical outputs from MLIPs. Pun et al.^42^ directly combine traditional physics-based potentials as regularization terms, Ibayashi et al.^43^ employ Sharpness-Aware Minimization for improved robustness, and Fu et al.^44^ apply Spectral Norm Regularization with Morse potential constraints. Pretraining on large data sets increases the structural diversity seen during training and thus enhances OOD performance. Maheshwari et al.^45^ demonstrate that OC20 pretraining enables extended stable simulations; Qi et al.^32^ developed DIRECT sampling for the Materials Project to create efficient pretraining data sets; and Takamoto et al.^46^ use Taylor expansion in data augmentation to generate large weakly labeled data sets. Other approaches include classical force field pretraining,^47^ Gardner et al.’s^48^ rattle-relax-repeat data augmentation followed by knowledge distillation, and Cui et al.’s^49^ iterative framework combining pseudolabeling with MD simulation feedback. Jung^50^ shows that pretraining and transfer learning improve atomic energy accuracy in neural network potentials; Jung and Cheng^51^ extend this strategy with effective charge separation to model nonequilibrium dynamics of ionic solids; Matin et al.^52^ apply ensemble knowledge distillation to compress multiple teacher potentials into efficient student models; and Amin et al.^53^ distill foundation models into fast, specialized force fields using energy Hessians.

Despite these progress, several limitations hinder widespread adoption of robust MLIPs for MD simulations. Active learning methods, though effective at improving accuracy in targeted regions, suffer from prohibitive computational costs due to iterative retraining and expensive ab initio calculations. Physics-informed and regularization approaches, while computationally more tractable, struggle to balance incorporating sufficient physical constraints with maintaining the model flexibility needed to capture complex potential energy surfaces accurately. These limitations motivate the development of alternative approaches that improve MLIP reliability without excessive computational overhead while providing robust mechanisms to prevent unphysical behaviors in MD simulations.

A complementary line of work targets MLIP robustness from the perspective of long-range physics. Several architectures augment MLIPs with explicit long-range terms: environment-dependent charge models and charge equilibration schemes,^54–57^ and Ewald-style Fourier-space message passing^58^ all extend the effective interaction range while preserving differentiability. These methods address a distinct failure mode from the one targeted in this work: long-range architectures correct for missing physics beyond the cutoff, whereas our focus is on short-range instabilities arising from inadequate repulsion and coordination-change physics in regions of configurational space underrepresented by equilibrium-biased training sets, a failure mode highlighted by recent benchmarks.^34,59^ Because our pretraining acts on the MLIP during training rather than modifying its architecture, it is in principle compatible with long-range-augmented backbones, and the two strategies can be combined.

In this work, we address this challenge through three key contributions. First, we demonstrate how traditional empirical potentials can enhance MLIPs by injecting physical knowledge through a pretraining-finetuning workflow. Second, we introduce two novel benchmarking metrics designed to detect unphysical behaviors in MLIP-generated MD trajectories in addition to traditional MLIP benchmarks on MD simulations and thus evaluate MLIP reliability. Third, through experiments with three representative MLIPs and benchmark data sets using our proposed suite, we demonstrate that physics-informed pretraining consistently improves MLIP reliability for MD simulations and provide theoretical and empirical analysis of the physical knowledge transfer mechanism.

## 2 Methodology

### 2.1 Machine Learning Interatomic Potentials

We evaluate our physics-informed pretraining framework and trajectory physicality benchmarking suite against three representative MLIP architectures that embody distinct design philosophies: Crystal Graph Convolutional Networks (CGCNN),^60^ M3GNet^17^ and TorchMD-NET.^61^ CGCNN is a graph neural network for materials that encodes atomic structures as crystal graphs with atoms as nodes and edges representing bonds. It iteratively updates atomic representations through graph convolutional operations that aggregate information from neighboring atoms. This model is invariant to translations, rotations, and permutations of the atoms in the system.^60^ M3GNet is an invariant MLIP that incorporates three-body interactions to achieve high accuracy across diverse materials systems. The model encodes atomic structures in a way that incorporates both distance and angular information, which enables it to better learn complex many-body interactions. M3GNet demonstrates exceptional transferability across different chemical compositions and structural motifs and is thus effective for materials discovery and property prediction tasks.^17^ TorchMD-NET is an equivariant transformer model which represents a further jump in complexity over the previous two examples. This model is equivariant to translations and rotations of the atoms and incorporates an attention based mechanism to further improve model expressivity.^61^ The selection of these three architectures allows us to assess the transferability of our proposed methods across different MLIP design paradigms and evaluate how our physics-informed pretraining and benchmarking approaches perform across distinct representation learning frameworks.

#### 2.1.1 Data Sets

We evaluate our methodology across three data sets of different chemical systems: Phosphorus, Silica and MPTrj subset. The Phosphorus data set comprises 4,798 structural configurations of phosphorus across multiple phases and morphologies. It includes GAP-RSS configurations from iterative random sampling, liquid structures (both molecular *P*_4_ and network phases), two-dimensional materials like phosphene with defects, bulk crystals at ambient and high pressures up to 100 GPa, and isolated molecular fragments. The structural diversity and inclusion of both stable and metastable configurations across the wide chemical space provides a stringent benchmark for evaluating MLIP transferability and accuracy. All structures are labeled with total energy and forces per atom by DFT computations.^62^ The Silica data set consists of 2864 amorphous and crystalline silica structures with liquid, glass, and crystalline phases, including distorted crystalline configurations, isolated dimers, and melt-quench trajectories. The data set is constructed by iteratively expanding a reference database generated through multiple cycles of GAP fitting and molecular dynamics simulations and is subsequently labeled with single-point DFT computation of total energy, forces per atom and cell stress.^63^ The MPTrj subset is a curated data set sampled from the Materials Project Trajectories (MPTrj) database,^64^ which contains relaxation trajectories for tens of thousands of crystal structures computed with DFT. Our subset consists of 4965 structures extracted from the MPTrj data set that contain exclusively lithium (Li), manganese (Mn), oxygen (O), and phosphorus (P) as constituent elements. The MPTrj subset provides a more complicated case for MLIP performance on materials systems, as it contains structures with more types of atoms than the silica and phosphorus data set, varying degrees of structural disorder and different oxidation states of metallic elements. The presence of multiple ionic species with varying coordination environments—ranging from highly ionic to van der Waals interactions—creates a demanding testbed for testing the transferability and stability of machine learning force fields.

### 2.2 Physics-Informed Pretraining Framework

#### 2.2.1 Leveraging Physics-Based Empirical Potentials

Although traditional empirical interatomic potentials have inferior accuracy to quantum mechanical methods due to their fixed functional forms, they encode fundamental physical principles that purely data-driven MLIPs often struggle to learn consistently from finite training data sets. Most critically, with their functional forms, traditional interatomic potentials generally satisfy constraints at limits of coordinates in that they predict energy *E*(*r* = 0) → ∞ and *E*(*r* → ∞) = 0,^65^ though different potentials achieve the constraints with different forms. Therefore, empirical potentials naturally incorporate short-range repulsive interactions that prevent unphysical atomic overlap, a behavior governed by the Pauli exclusion principle^66^ that MLIPs often violate (as shown in Figure 1). This shortcoming stems from the compositional bias in most material data sets: they are dominated by structures near equilibrium and lack examples with atoms at short separations.

**Figure 1:**
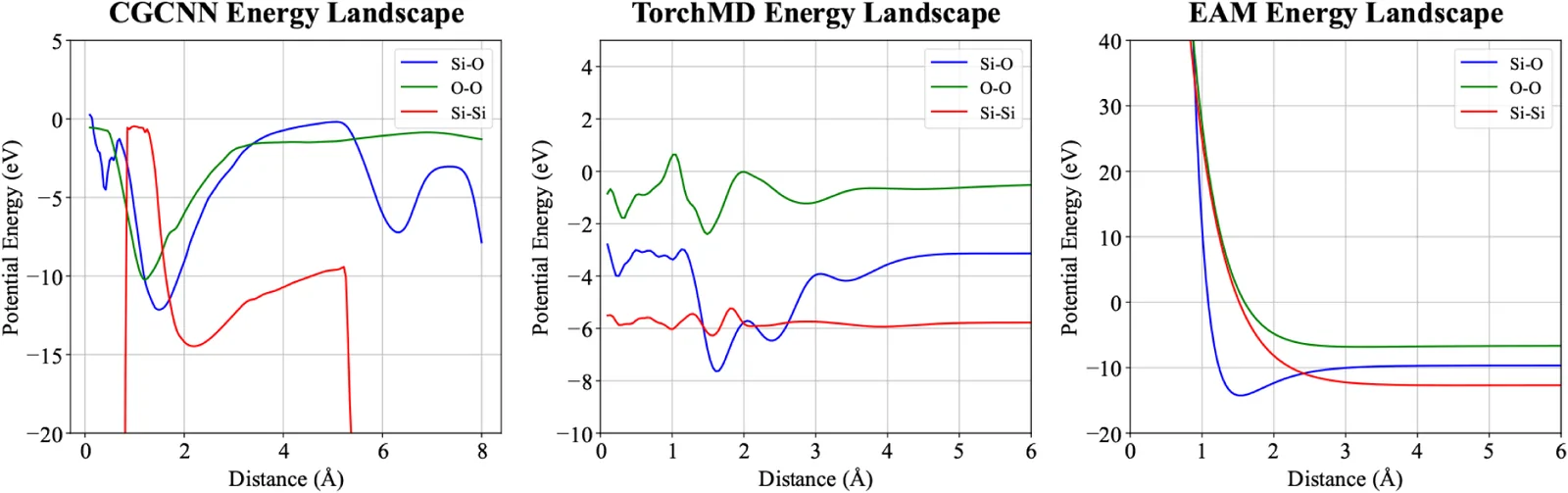
Predicted energy landscapes from MLIP CGCNN and TorchMD, and the EAM potential for energy prediction of three types of interactions in the Silica data set. Clear unphysical patterns are observed in both MLIPs below 1Å and above 6Å for CGCNN.

Additionally, traditional potentials generally produce well-shaped, smooth energy landscapes with physically meaningful curvature, which ensures stable force calculations and prevents the erratic energy fluctuations that can lead to simulation instabilities. By transferring this embedded physical knowledge into MLIPs, we hypothesize that the resulting models will exhibit enhanced robustness and improved adherence to fundamental physical constraints, particularly in unexplored regions of configurational space where pure machine learning approaches typically fail.

#### 2.2.2 Implementation of the Empirical Potential

The Embedded Atom Method (EAM)^67^ is an empirical method for computing potential energy surfaces commonly for metallic systems. Although modern MLIPs significantly outperform EAM in predicting forces and energies, EAM maintains several crucial advantages that justify its inclusion for improving stability of MLIP-empowered MD simulation. EAM potentials require only a few dozen fitted parameters—orders of magnitude fewer than MLIPs, which typically require millions—and their fixed functional forms inherently respect physical principles. The robustness and physical grounding of EAM are properties we exploit in this work. With EAM, the total energy of a system is expressed as the sum of two distinct contributions: an embedding energy term that represents the energy required to place an atom into the electron density created by all surrounding atoms and a pairwise interaction term between each pair of atoms in the system. We implement this formulation with an additional atomic energy term (eq 1), which extends the standard EAM approach and is detailed below. In eq 1, *F*_α_ represents the energy embedding function,^67^ ρ_β_ denotes the electron density function, and *V*(*r*) represents the pairwise interaction function. In the following sections, we elaborate on the implementation details of the three components of the eq 1.

1 $$E_{tot}=F_{\alpha }\left(\underset{i\neq j}{\sum }\rho_{\beta }\left(r_{ij}\right)\right)+\frac{1}{2}\underset{i\neq j}{\sum }V\left(r_{ij}\right)+E_{atom}$$

The determination of accurate energy embedding and electron density functions is challenging due to the complex quantum mechanical nature of electron densities in metals. While first-principles methods such as density functional theory (DFT)^10,11^ could theoretically provide precise electron densities, their computational expense renders them impractical for large-scale applications. Following prior work,^68^ we employ multiple exponential decay functions to approximate the electron density term and adopt a simple quadratic form for the energy embedding function. Our electron density construction (eq 2) employs learnable parameters: $A,\beta \in R^{n_{a}\times n_{b}}$ and $B,\rho_{0}\in R^{n_{a}}$ , where *n*_*a*_ is the number of diatomic interaction types in the data set and *n*_*b*_ is the number of exponential basis functions employed.

2 $$\begin{array}{ll} \rho_{\beta }\left(r_{ij}\right) & =\overset{n_{b}}{\underset{k=1}{\sum }}A_{k}\cdot \exp\left(-\beta_{k}\cdot r_{ij}\right)\\ F_{\alpha }\left(\rho \right) & =B\cdot {\left(\rho -\rho_{0}\right)}^{2},\mathrm{with}\rho =\underset{j\neq i}{\sum }\rho_{\beta }\left(r_{ij}\right) \end{array}$$

For the pairwise interaction term (the second term in eq 1), we use the Morse potential,^69^ an empirical function that describes interatomic bonding energy as a function of distance. Unlike harmonic models, the Morse potential captures anharmonicity and realistic dissociation behavior.

3 $$V\left(r\right)=D_{e}{\left(1-e^{-a(r-r_{e})}\right)}^{2}$$

For two atoms separated by distance *r*, the Morse potential energy *V*(*r*) is given by eq 3. The learnable parameters are $D_{e},a,r_{e}\in R^{n_{a}}$ , where *D*_*e*_ is the well depth (dissociation energy), *r*_*e*_ is the equilibrium bond distance, and *a* controls the width of the potential well. This compact, physically meaningful parametrization captures both anharmonicity and dissociation behavior.

Finally, we add an atomic energy term *E*_*atom*_ as a learnable per-element reference that accounts for isolated atom properties and sets a physically meaningful zero-point. This improves prediction of absolute and formation energies across chemical environments and is implemented as a per-element scalar learned alongside other EAM parameters.

We optimize all EAM parameters using backpropagation with the AdamW optimizer to minimize the Mean Absolute Error (MAE) of energies, forces, and stress. All components—electron density coefficients (*A*, β, *B*, ρ_0_), Morse parameters (*D*_*e*_, *a*, *r*_*e*_), and per-element atomic energies—are treated as learnable variables. The equilibrium distance *r*_*e*_ is initialized to the sum of covalent radii for each pair type (a physically meaningful starting point), while all other parameters are initialized to 1. This initialization strategy ensures that the optimization begins from a chemically reasonable configuration while allowing the model to adaptively learn optimal parameter values through gradient-based training on the reference data.

Figure 2 visualizes the decomposition of the trained EAM potential for the silica data set across the three primary interaction types. The results show that while the Morse potential captures fundamental pairwise bonding, the electron density term provides corrections that significantly reduce the combined potential’s EFS MAE relative to either component alone. The trained EAM potential successfully reproduces the expected chemical behavior of silica: strong Si–O bonding with a potential minimum near 1.6 Å, consistent with experimental Si–O bond lengths in SiO_2_ ^70^ and weaker O–O and Si–Si interactions. This decomposition demonstrates that the EAM framework provides a physically interpretable foundation for enhancing the stability of MLIP-enabled MD simulations.

**Figure 2:**
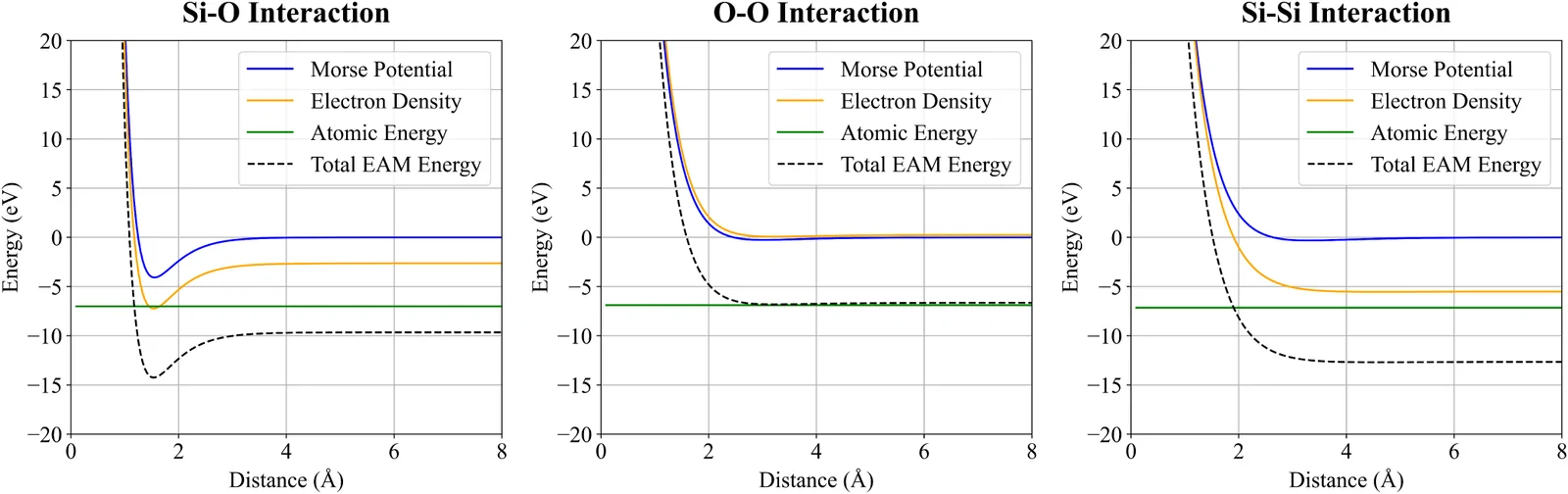
Decomposition of the trained EAM potential for modeling three types of diatomic interaction in the Silica data set with individual energy components as a function of interatomic distance. The plots display the contribution of electron density (orange solid line), Morse pairwise interaction (blue solid line), atomic energy (green solid line) and total EAM energy (black dashed line) for the three primary atom pair types in silica: (left) Si–O interactions, (center) O–O interactions, and (right) Si–Si interactions. All energies are shown relative to the cutoff radius of 8.0 Å.

### 2.3 Effectiveness of the EAM Potential

Figure 3 demonstrates the effectiveness of our fitted EAM potential across diverse material systems. The fitted potential energy curves exhibit physically reasonable behavior: appropriate short-range repulsive walls, well-defined energy minima, and smooth asymptotic decay at large distances. Across all three data sets (silica, phosphorus, and MPTrj subset), the EAM potential successfully reproduces the characteristic bonding patterns and energy scales of each system. The EAM formulation successfully models diverse bonding types, from covalent Si–O interactions in silica to metallic bonding in transition metals. Although the EAM potential lacks DFT’s quantitative accuracy, as seen in the imperfect well location for MPTrj subset interactions (e.g., 2.16 – 2.30 Å for Mn–O, 2.13 – 2.36 Å for Li–O, 1.53 Å for P–O, according to a typical LiMnPO_4_ structure mp-18997 in the materials project), it captures the fundamental physics needed to generate physically meaningful pretraining data. This data biases the MLIP toward chemically sensible solutions and empirically improves the physicality of MLIP-generated MD trajectories.

**Figure 3:**
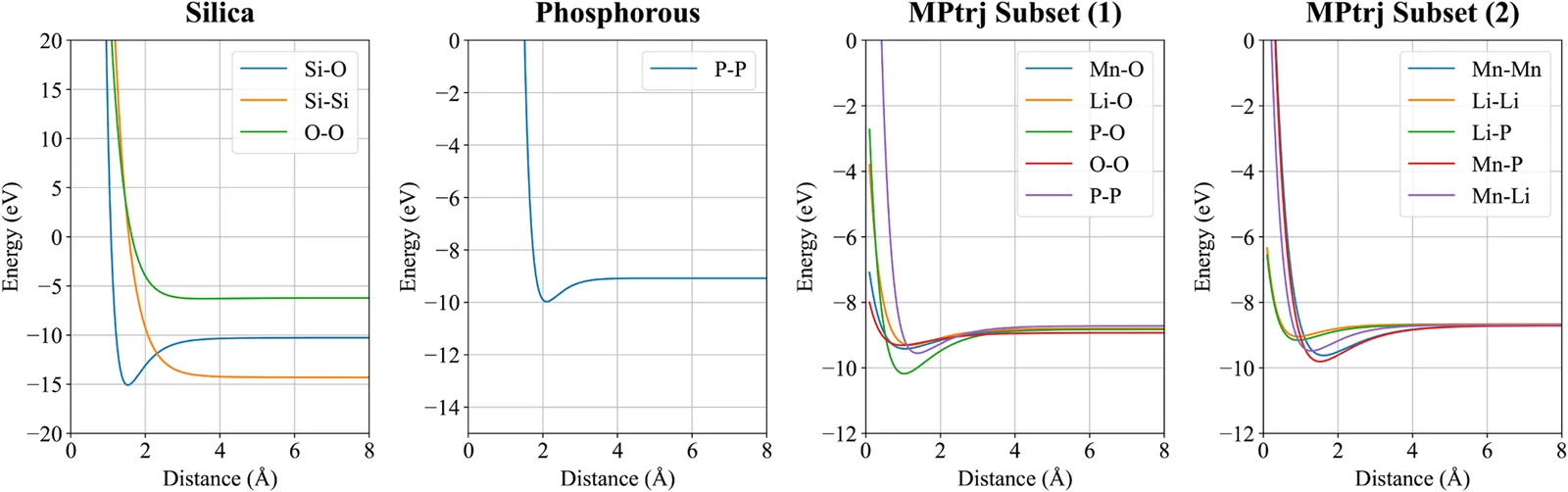
EAM potential energy curves for key interatomic interactions across three benchmark data sets. The plots show the fitted EAM total energy as a function of interatomic distance for the most chemically significant atom pair interactions in each system: (left) Si–O, O–O and Si–Si interactions in the silica data set, (center left) P–P interactions in the phosphorus data set, and (center right and right) all interactions in the MPTrj subset. Five interactions are shown per plot for clarity.

### 2.4 Physics-Informed Pretraining Workflow

We implement a systematic pretraining-finetuning pipeline (Figure 4) that leverages EAM to enhance MLIP robustness. The approach has two phases: pretraining on a data set generated with EAM-labeled perturbed structures, and finetuning on original quantum mechanical data.

**Figure 4:**
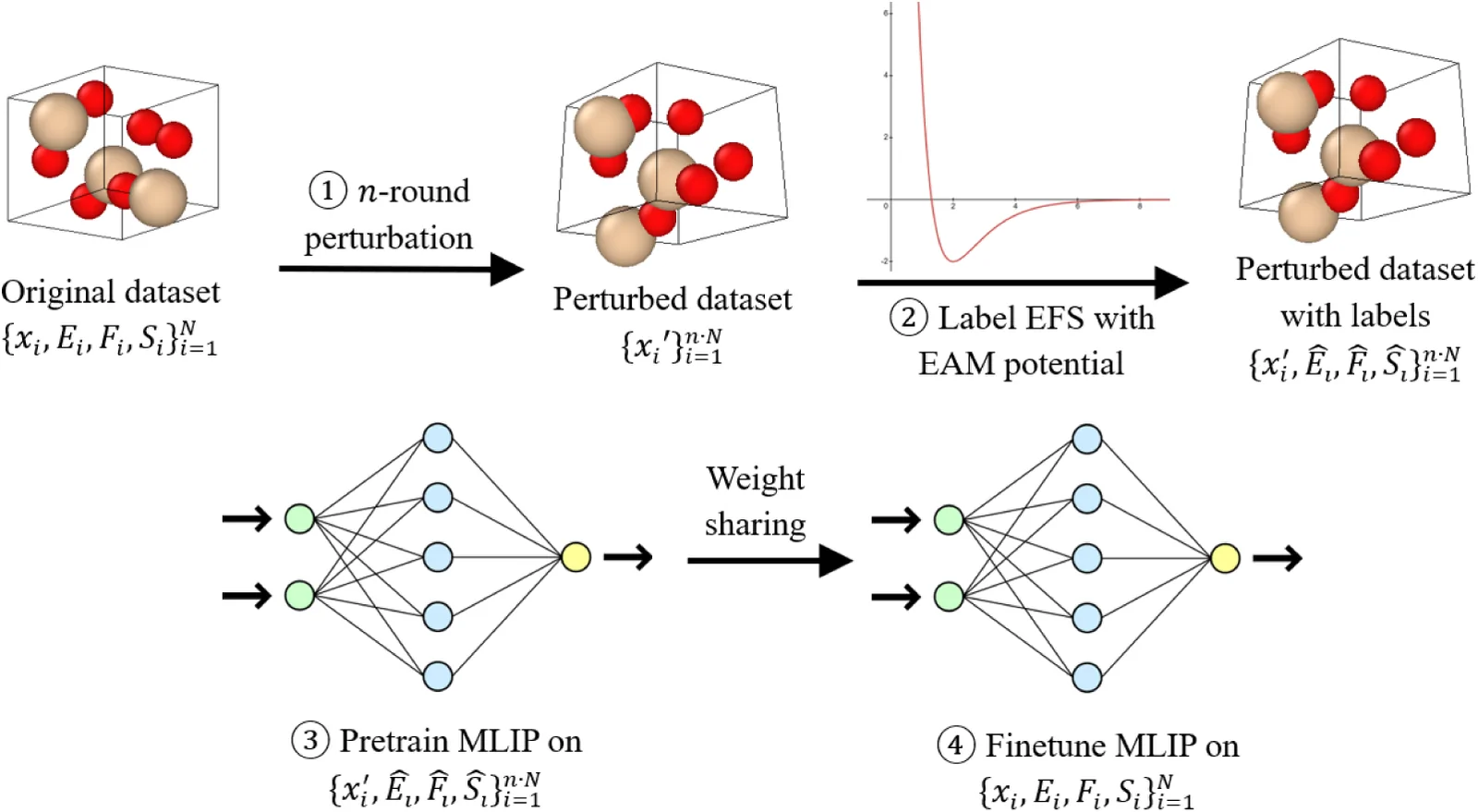
Physics-informed pretraining workflow. Starting with the original quantum mechanical data set, atomic positions are systematically perturbed using the two-step algorithm to generate diverse configurations. The perturbed structures are labeled using the trained EAM potential. The MLIP is pretrained on the EAM-labeled data, then finetuned on the original quantum mechanical data via weight sharing.

#### 2.4.1 Pretraining Data Set Construction

We generate the pretraining data set by systematically perturbing atomic positions in the original quantum mechanical reference structures. This augmentation strategy explores diverse configurations while ensuring physical consistency through EAM-based labeling. For each reference structure $P\in R^{N_{a}\times 3}$ , we generate perturbed configurations $P^{\prime }\in R^{N_{a}\times 3}$ using a two-step algorithm (Algorithm 1) that combines targeted compression with random perturbation. The algorithm identifies close atomic pairs and reduces their separation distances, then applies random perturbations to selected atoms. This creates configurations with both coordination changes and structural distortions. Each perturbed structure is labeled with energies, forces, and stresses using the trained EAM potential. The dual perturbation strategy with systematic compression for coordination changes and random displacement for structural distortions provides physically consistent reference data that enables robust interpolation across diverse configurations.

#### 2.4.2 Pretraining and Finetuning

The backbone MLIP is initially trained on the EAM-labeled data set. This pretraining establishes physically informed initialization and guides the model toward solutions consistent with the physics encoded in EAM. Following pretraining, we finetune the model on the original quantum mechanical data initializing the MLIP with the pretrained weights while making all parameters tunable in finetuning. Finetuning preserves the physical intuition from pretraining while achieving ab initio accuracy. The resulting MLIP is evaluated via MD simulation benchmarking.
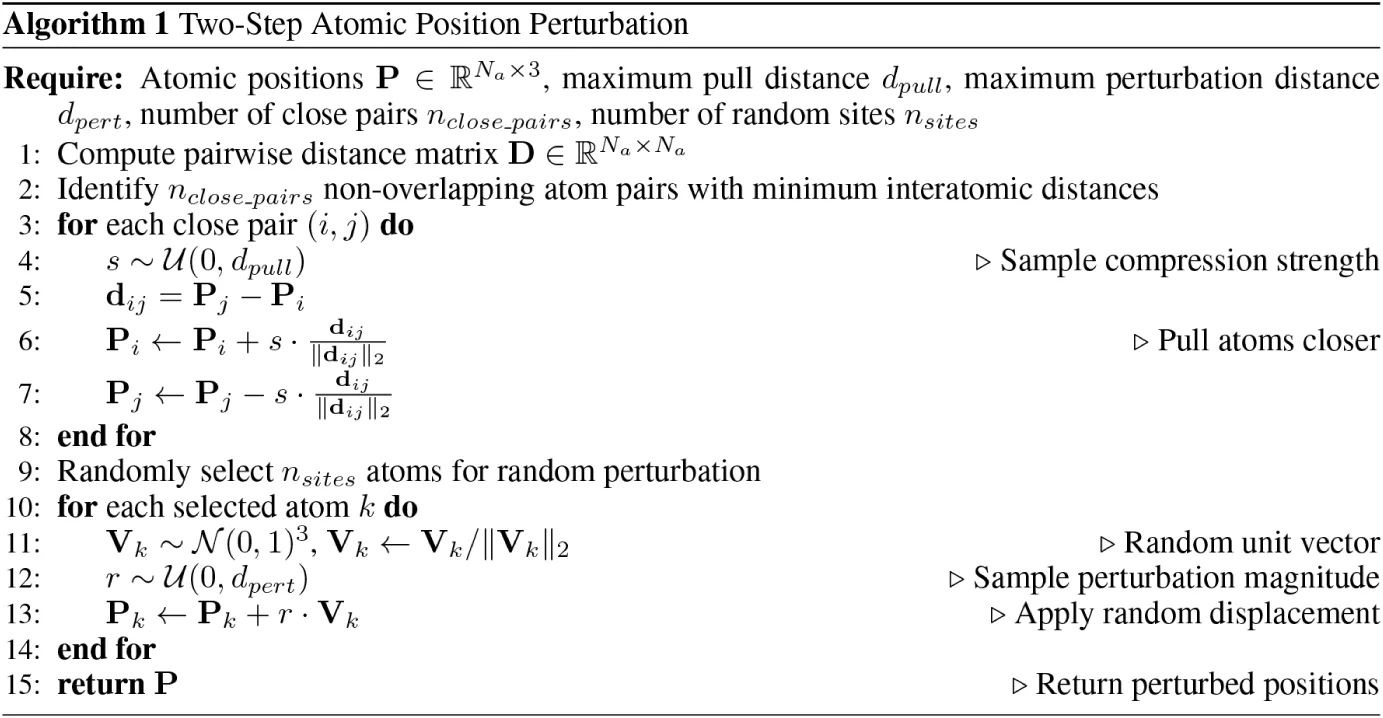


### 2.5 Benchmarking Suite for Trajectory Physicality

#### 2.5.1 Molecular Dynamics Simulations

Molecular dynamics simulations are used to evolve the state of an atomistic system with Newtonian equation of motion given the force acting on each atom at time *t*. With *T* steps, the simulation produces a trajectory of positions $J=\{P_{t}\in R^{N\times 3}{\}}_{t=1}^{T}$ , in which **P**_*t*_ represents the positions of atoms at time step *t*. In this project, we primarily leverage NVT and NPT simulation with Langevin and Berendsen dynamics, respectively.

#### 2.5.2 Metrics for MD Trajectory Physicality

To quantitatively describe the quality of MLIP-generated MD trajectories, existing studies^37,41,71^ typically rely on system observables such as radial distribution functions (RDF), diffusion coefficients, and vibrational density of states by comparing these properties between MLIP and ab initio trajectories. Although these benchmarks assess how well MLIPs reproduce ab initio data, they do not directly detect unphysical artifacts or physical law violations within the trajectories. To address this gap, we introduce two structural metrics to detect unphysical abnormalities: overlapping atoms and structural collapse.

Given a trajectory of *T* timesteps *J* = {*A*_0_, ..., *A*_*T*_}, where each *A*_*i*_ represents the structural configuration at the *i* ^*th*^ time step, our benchmarking suite consists of the following two physicality metrics:

##### 2.5.2.1 Overlapping Atoms

This metric detects catastrophic failures when atoms approach unphysically close distances, violating fundamental atomic principles. Such overlaps indicate inadequate repulsive force representation in the MLIP.

A trajectory is classified as unphysical if, at any time step, there exists a pair of atoms whose separation distance falls below a dynamically determined threshold:

$$\mathrm{Trajectory}\;J\;\mathrm{is}\;\mathrm{unphysical}\Leftrightarrow \exists k\in 1,...,T,\underset{i\neq j}{\min}|\left(P_{A_{k}}\right)i-(PA_{k}{)}_{j}{|}_{2}\leq \mathrm{threshold}$$

To accommodate compositional diversity, we define an adaptive threshold based on the initial equilibrium structure. This approach ensures sensitivity to structural violations while respecting the characteristic length scales of different materials.

$$\mathrm{threshold}=\frac{1}{2}\cdot \min(d_{ij})$$

##### 2.5.2.2 Lindemann Index

The Lindemann index quantifies thermal disorder and structural stability by measuring atomic positional fluctuations relative to equilibrium.^72^ Originally developed to characterize melting, the Lindemann index detects excessive structural mobility that indicates simulation artifacts or unphysical dynamics.

For a system with *n* atoms, the local Lindemann index *q*_*i*_ quantifies the normalized RMS bond length fluctuation for atom *i*,^73^ while the global index *q*_global_ is the system-wide average, where ⟨·⟩ denotes temporal averaging:

$$q_{i}=\frac{1}{N-1}\underset{j\neq i}{\sum }\frac{\left\langle r_{ij}^{2}\right\rangle -{\left\langle r_{ij}\right\rangle }^{2}}{\left\langle r_{ij}\right\rangle },,q_{global}=\frac{1}{N}\underset{i}{\sum }q_{i}$$

We use the Lindemann index to detect excessive structural reorganization beyond physically reasonable thermal motion. A trajectory is unphysical if any atom shows anomalously large fluctuations (local instability) or the system exhibits collective instability. Since melting occurs at Lindemann indices of 0.1–0.15,^74^ we conservatively set thresholds at 0.25 (local) and 0.2 (global) to detect instabilities while avoiding false positives from thermal motion:

$$\mathrm{Trajectory}\;J\;\mathrm{is unphysical}\Leftrightarrow \exists i:q_{i}>0.25\mathrm{or}q_{\mathrm{global}}>0.2$$

## 3 Results

This section presents experimental evidence for the effectiveness of our approach in developing stable MLIPs for MD simulations. We first evaluate how pretraining on EAM-labeled data improves MLIP performance in empowering physically reliable MD simulation with metrics proposed in Section 2.5 and how MLIP trajectories compare to DFT trajectories. We also evaluate EFS (energy, forces, stress) prediction accuracy to confirm that our method preserves structural property accuracy without compromising prediction quality.

### 3.1 Experimental Setup

We evaluate our physics-informed pretraining approach across three diverse data sets and three MLIP architectures: CGCNN, TorchMD-NET, and M3GNet (introduced in Sections 2.1). Because TorchMD-NET and M3GNet outperform CGCNN at baseline, we implement a stricter protocol for these two architectures by deliberately limiting training data, including EAM fitting data. This constraint creates a challenging scenario that better demonstrates the value of physics-informed pretraining when data is limited.

### 3.2 Physicality of MLIP-Generated MD Trajectories

To evaluate our physics-informed pretraining under realistic conditions, we conducted comprehensive MD benchmarking across all three data sets. For each data set, we randomly sampled 100 test structures and performed 5000-step simulations under different thermodynamic ensembles and temperatures. MD simulations test model robustness by requiring the MLIP to maintain physical consistency over extended trajectories while exploring configurations beyond the training distribution. We compared our method against baseline MLIPs and MLIPs trained with Sharpness Aware Minimization^43^ (SAM) with stability metrics from Section 2.5.2: overlapping atoms and Lindemann index. We included SAM to benchmark our physics-informed pretraining approach against a representative regularization-based strategy for improving MLIP robustness. SAM was specifically selected as a representative for all regularization-based methods because it is a well-known, lightweight, easy-to-implement and model-agnostic technique that has been specifically applied to improve MLIP stability in prior work,^43^ so that it is a natural and fair point of comparison that requires no additional data, no auxiliary models, and no architectural modifications, while targeting the same goal of improved MD trajectory stability. All results are presented in Table 1.

**Table 1:** Trajectory Stability Assessment for Hybrid MLIPs Across Diverse Datasets and Simulation Conditions^a^

Data set	MD Setting	Setup	Atom Overlap	Lindemann Index
Silica (full)	NVT (2000K, 2 fs)	CGCNN	5	2
		CGCNN + SAM	**4**	4
		**CGCNN + Pretrain**	**4**	**0**
	NPT (2000K, 2 fs)	CGCNN	9	7
		CGCNN + SAM	8	11
		**CGCNN + Pretrain**	**5**	**2**
Silica (10%)	NVT (2000K, 2 fs)	TorchMD	15	8
		TorchMD + SAM	12	8
		**TorchMD + Pretrain**	**5**	**7**
		M3GNet	**2**	3
		**M3GNet + Pretrain**	**2**	**2**
	NPT (2000K, 2 fs)	TorchMD	14	7
		TorchMD + SAM	8	8
		**TorchMD + Pretrain**	**5**	**6**
		**M3GNet**	**2**	**2**
		**M3GNet + Pretrain**	**2**	**2**
Phosphorus (full)	NVT (1250K, 1 fs)	CGCNN	35	70
		CGCNN + SAM	34	**64**
		CGCNN + Pretrain	**22**	65
Phosphorus (5%)	NVT (1250K, 1 fs)	TorchMD	9	42
		TorchMD + SAM	25	57
		**TorchMD + Pretrain**	**6**	**24**
		M3GNet	**0**	43
		**M3GNet + Pretrain**	**0**	**4**
MPTrj Subset (full)	NVT (500 K, 1 fs)	CGCNN	89	39
		CGCNN + SAM	7	5
		**CGCNN + Pretrain**	**2**	**4**
	NPT (250 K, 1 fs)	CGCNN^†^	*	*
		CGCNN + SAM	8	9
		**CGCNN + Pretrain**	**4**	**6**
MPTrj Subset (10%)	NVT (1000K, 1 fs)	TorchMD	70	98
		TorchMD + SAM	49	97
		**TorchMD + Pretrain**	**11**	**40**
		M3GNet	100	100
		**M3GNet + Pretrain**	**0**	**19**
	NPT (750 K, 1 fs)	TorchMD	84	98
		TorchMD + SAM	28	72
		**TorchMD + Pretrain**	**1**	**16**
		M3GNet	100	100
		**M3GNet + Pretrain**	**0**	**82**

a All Molecular Dynamics Simulations Were Conducted for 5000 Timesteps on 100 Randomly Sampled Structures from Each dataset’s Test Set. Values Represent the Number of Trajectories (Out of 100) Exhibiting Unphysical Behavior as Detected by Atom Overlap and Lindemann Index Criteria. NVT and NPT Simulations Were Performed with Langevin and Berendsen Dynamics Using the Atomic Simulation Environment (ASE) Package. NPT Simulations Were Not Performed on the Phosphorous Dataset Due to the Absence of Stress Labels and NPT’s Requirement for Stress Tensors.

The magnitude of improvement, however, varies across the configurations in Table 1, and this variation is itself informative. The largest gains are observed for models trained on MPTrj, which contains only relaxation trajectories and therefore exposes baseline models to a narrow region of the PES; such models are essentially unable to handle the configurations encountered during finite-temperature MD, which leaves substantial room for pretraining to contribute. In contrast, the phosphorus data set^62^ was deliberately constructed with diverse configurations including GAP-RSS samples, liquid and amorphous phases, defects, and high-pressure structures up to 100 GPa, so baseline models trained on it already cover a substantial portion of the relevant PES and exhibit correspondingly smaller improvements from pretraining. On the architectural side, CGCNN is less expressive than the equivariant transformer TorchMD-NET or the three-body M3GNet, so its baseline failures may stem in part from limited model capacity rather than purely from out-of-distribution generalization, which pretraining is designed to address; conversely, only 10% and 5% of the training set are randomly sampled for TorchMD-NET and M3GNet to create a low-data regime in which pretraining provides the greatest benefit. Taken together, these observations support a coherent picture that physics-informed pretraining is most effective when baseline models have insufficient PES coverage to handle MD-explored configurations. Rather than indicating inconsistent performance, the variation across systems demonstrates that the method targets the specific failure mode it was designed to address, namely the gap between the training distribution and the configurations encountered during MD simulation.

### 3.3 Capability of MLIPs to Recover DFT MD Trajectories

To complement the physicality benchmarking, we assess MLIP performance by comparing structural properties with reference DFT MD simulations. We conducted DFT Langevin NVT simulations on ten representative MPTrj Subset structures under identical conditions as the corresponding MLIP simulations. We use Radial Distribution Functions (RDFs) as a quantitative structural metric, following prior work.^71^ RDFs capture the statistical distribution of interatomic distances and reveal structural ordering. By comparing RDFs from DFT and MLIP trajectories, we evaluate how well MLIPs reproduce structural dynamics and correlation patterns. Figure 5 shows RDF analysis for four representative atomic pair types: Li–O, Mn–O, O–O, and Mn–P. The first two represent typical coordination environments with strong chemical bonding, while the latter two represent nonbonded interactions revealing medium-range structural correlations.

**Figure 5:**
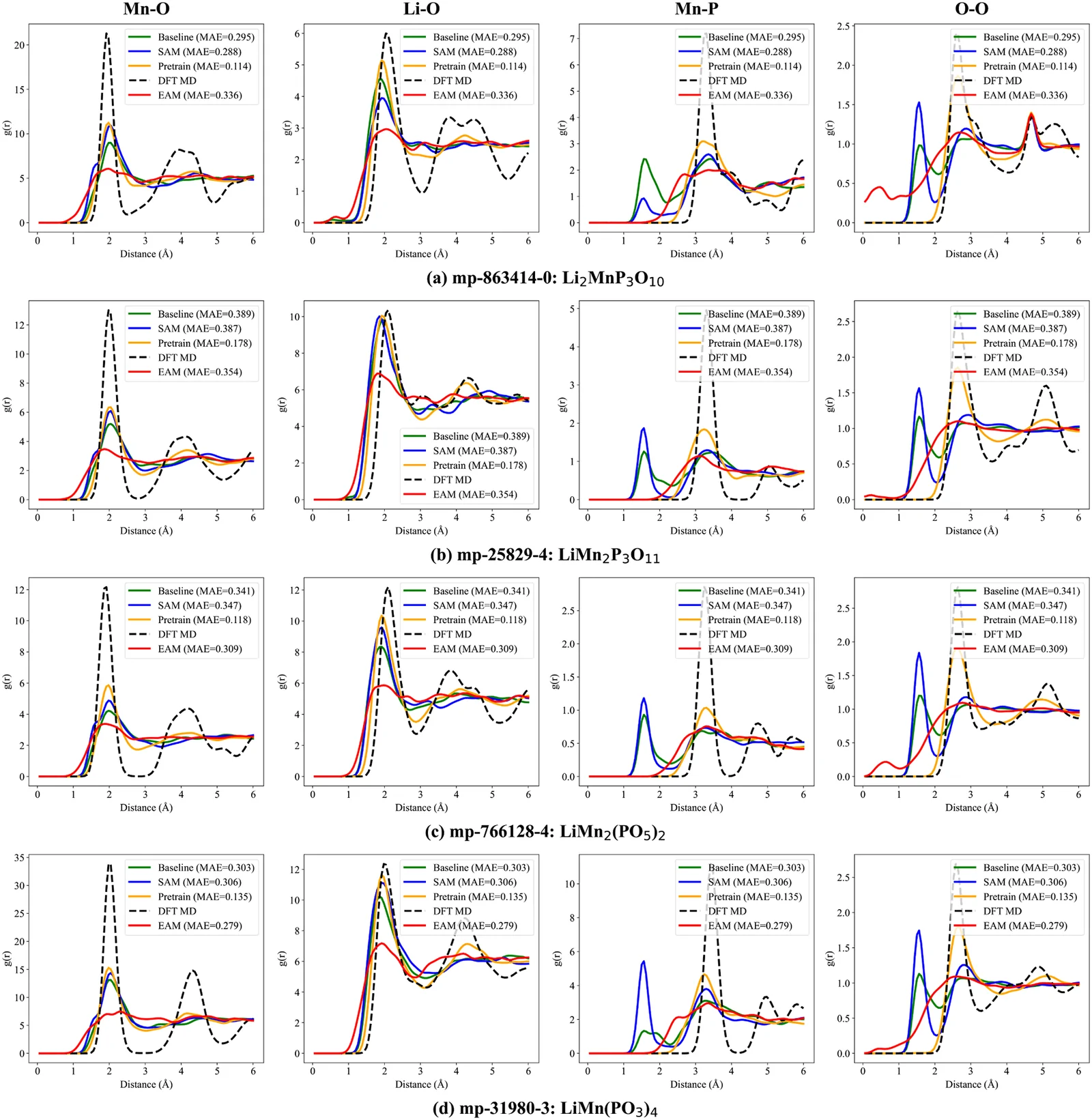
Radial distribution function comparison between DFT molecular dynamics and TorchMD predictions for four representative MPTrj Subset structures. The plots show RDFs for four atomic pair interactions (Mn–O, Li–O, Mn–P, O–O) across four selected structures: (a) mp-863414–0, (b) mp-25829–4, (c) mp-766128–4, and (d) mp-31980–3. Black dashed lines represent reference DFT MD trajectories, while colored lines show MLIP predictions using different training approaches: orange curves show our proposed pretraining method, blue curves show SAM optimization results, green curves represent the baseline vanilla TorchMD model, and red curves represent the EAM potential as a standalone potential.

The RDF results demonstrate that physics-informed pretraining substantially improves modeling of diverse atomic interactions across the MPTrj Subset structures, with notable enhancements for both bonded and nonbonded pairs. The pretrained TorchMD model (orange lines) consistently achieves superior agreement with DFT reference trajectories (black dashed lines) compared to both SAM optimization (blue lines) and baseline TorchMD (green lines) across all interaction types. For bonded interactions (Mn–O and Li–O), our method reproduces the characteristic first coordination shell peaks around 1.5–2.5 Å and maintains peak intensities and shapes closely matching DFT reference data. More significantly, for nonbonded interactions (Mn–P and O–O), pretraining eliminates the unphysical artifacts present in both alternatives, which show anomalous short-distance peaks where strong repulsion should dominate and RDF values should be near zero. These improvements are physically meaningful: anomalous short-distance peaks indicate MLIPs predict chemically implausible configurations with unrealistic close contacts. Figure 5 demonstrates that the improvement in physicality comes directly from the EAM potential knowledge, as the standalone EAM potential’s RDF shows similar peak patterns to our model. However, the EAM potential exhibits larger MAE than other MLIPs due to its weaker capability in predicting energy and forces. The enhanced repulsive behavior at short distances aligns with the reduced atom overlap abnormalities in our trajectory physicality benchmarks (Section 3.2). Consistent performance across structures validates the robustness and transferability of our physics-informed approach for complex oxide materials.

### 3.4 Prediction Accuracy of MLIPs

We demonstrate that the proposed method preserves or improves MLIP prediction accuracy. We use energy, force, and stress (EFS) Mean Absolute Error (MAE) on each data set’s test set as benchmarking metrics; energy and forces MAE for the Phosphorus data set due to stress data availability. Table 2 presents a comprehensive comparison of prediction errors for baseline MLIP, SAM-optimized MLIP,^43^ and our EAM-assisted pretraining approach.

**Table 2:** Prediction Accuracy Comparison for MLIPs with and without EAM-Assisted Pretraining^a^

Data set	Setup	E Error	F Error	S Error	Total Error
Silica (full)	CGCNN	29.062	0.208	0.00290	10.796
	CGCNN + SAM	27.722	0.214	0.00233	11.071
	**CGCNN + Pretrain**	23.871	0.200	0.0127	**10.837**
Silica (10%)	TorchMD	8.157	0.117	0.00117	6.010
	**TorchMD + SAM**	5.071	0.107	0.000876	**5.460**
	TorchMD + Pretrain	7.820	0.113	0.000374	5.797
	M3GNet	3.252	0.114	0.00441	5.967
	**M3GNet + Pretrain**	4.069	0.112	0.00286	**5.781**
Phosphorus (full)	CGCNN	9.976	0.252	/	12.677
	CGCNN + SAM	10.778	0.265	/	13.338
	**CGCNN + Pretrain**	9.476	0.232	/	**11.709**
Phosphorus (5%)	**TorchMD**	16.301	0.201	/	**10.222**
	TorchMD + SAM	7.439	0.233	/	11.712
	TorchMD + Pretrain	22.196	0.202	/	10.336
	M3GNet	8.170	0.257	/	12.931
	**M3GNet + Pretrain**	23.033	0.248	/	**12.629**
MPTrj (full)	CGCNN	1.485	0.0914	0.00191	4.679
	CGCNN + SAM	3.518	0.0847	0.00180	4.359
	**CGCNN + Pretrain**	2.575	0.0782	0.00167	**4.017**
MPTrj (10%)	TorchMD	6.139	0.0931	0.00184	4.806
	**TorchMD + SAM**	4.910	0.0736	0.00150	**3.803**
	TorchMD + Pretrain	4.945	0.0881	0.00179	4.537
	M3GNet	3.065	0.0854	0.00231	4.416
	**M3GNet + Pretrain**	6.568	0.0652	0.00171	**3.412**

a Energy, Force, and Stress Errors are Reported as Mean Absolute Error (MAE) in eV, eV/Å, and GPa, Respectively. Total Error is a Weighted Combination Using Weights 0.01:50:50 for Energy:Force:Stress (0.01:50 for Phosphorous Dataset Due to Missing Stress Labels). Dataset Percentages Indicate the Fraction of Training Data Used. The “Setup” Column Compares Three Approaches: Baseline MLIP, SAM-Optimized MLIP, and Our Finetuned MLIP with Pretraining. Lowest Total Error for Each Dataset is Highlighted.

Our physics-informed pretraining approach proves largely nonintrusive: the finetuned MLIP maintains competitive accuracy with modest, consistent improvements over baselines. Across all data sets, pretraining always improves the predictive capabilities of the underlying MLIP architectures (besides TorchMD + Phosphorus, with almost the same performance), as shown in Table 2.

### 3.5 Knowledge Transfer from EAM Potential to MLIP

The effectiveness of physics-informed pretraining can be demonstrated qualitatively through visualization of knowledge transfer from EAM to the final finetuned MLIP (Figure 6). This analysis reveals how the pretraining-finetuning protocol improves physicality across a wider configurational space. We use the Silica data set for visualization due to its moderate interaction complexity. Knowledge transfer occurs through structured exposure to physical energy landscapes during pretraining. Figure 6(a) compares the finetuned model against a baseline trained only on the original data set. The finetuned model exhibits more physically reasonable potential curves, particularly in repulsive regions where limited training data typically causes poor extrapolation. This improvement stems from physics-informed initialization, which provides a chemically reasonable starting point for optimization.

**Figure 6:**
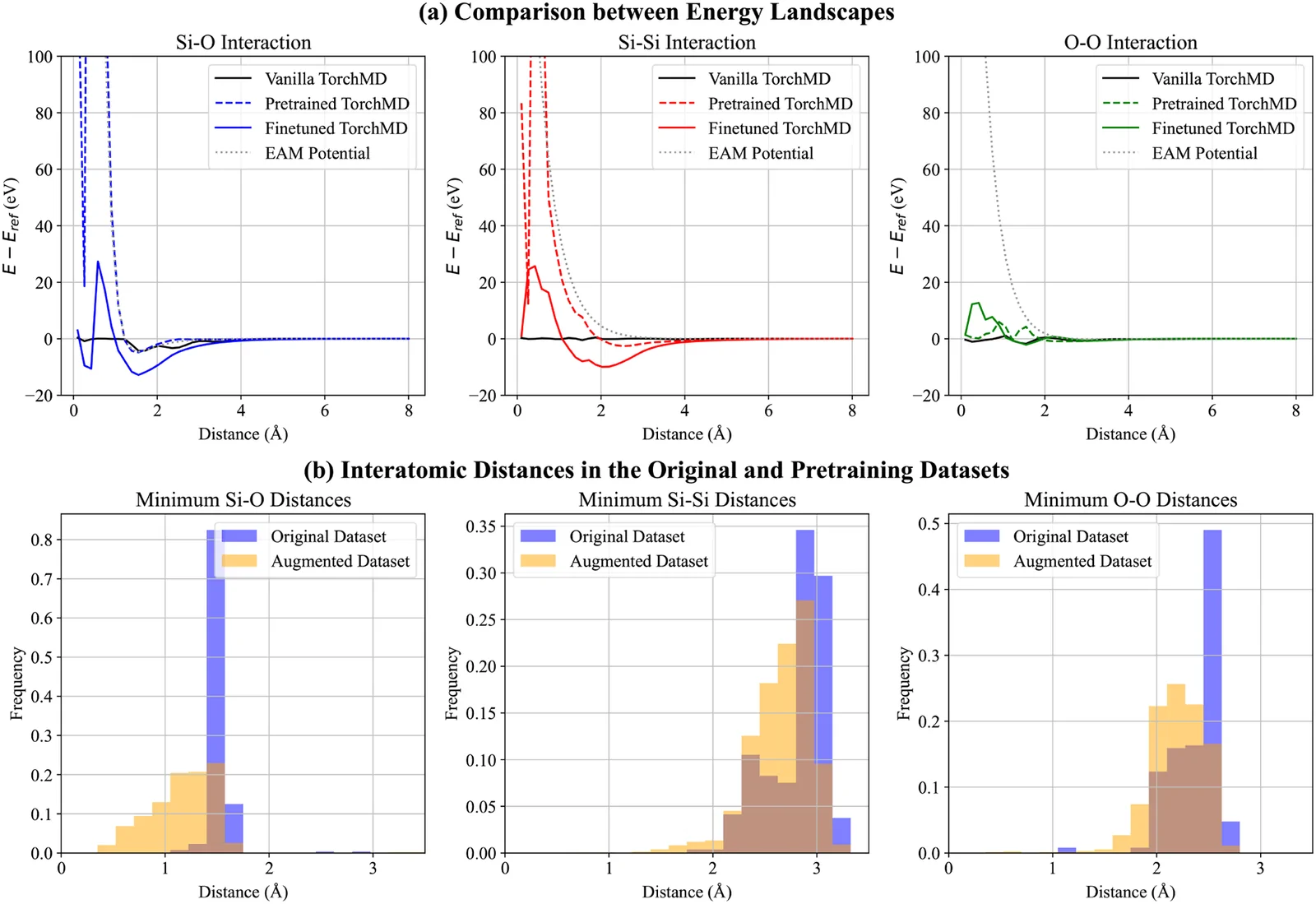
Knowledge transfer from EAM to MLIP. (a) Potential energy surface of the finetuned (colored solid lines), pretrained (colored dashed lines), the baseline model (black solid lines), and the trained EAM potential (gray dots). Energies for each model are shown relative to a reference energy, *E_ref_*, computed as the average predicted energy for pairs at large (greater than 7Å) interatomic distance (*r* → ∞). Since absolute energy is only defined up to an arbitrary constant, this shift allows direct comparison of well depths and repulsive behavior across models. The finetuned MLIP shows superior physicality for Si–O and Si–Si interactions, especially in repulsive regions where the baseline exhibits poor extrapolation, though improvement in physicality of O–O seems insignificant. (b) Minimum interatomic distance distributions: for each structure in the original and pretraining data sets, we compute the minimum Si–Si, O–O, and Si–O distances. The pretraining data set (orange) substantially expands coverage compared to the original data set (blue) significantly for Si–O pairs (0.5–1.5Å), moderately for Si–Si pairs (1.5–2.0Å) and relatively insignificant for O–O pairs. Pretraining includes compressed configurations underrepresented in DFT sets, enabling the MLIP to learn repulsive behavior during pretraining.

A critical advantage of our approach is the systematic expansion of configurational coverage. Figure 6(b) shows how the pretraining data set (orange) significantly broadens interatomic distance distributions compared to the original data set (blue) across all three interaction types: Si–O, Si–Si, and O–O. Our perturbation scheme systematically samples compressed configurations with shorter interatomic distances that are typically underrepresented in DFT training sets but frequently encountered during MD simulations. This expanded coverage enables the MLIP to learn physically consistent repulsive behavior across a broader configurational range using EAM-provided labels, and the finetuned MLIP consequently exhibits enhanced stability and reduced unphysical behavior during MD simulations. Yet, we observed that this expansion is nonuniform for each interaction type in the data set: coverage increases substantially for Si–O pairs (0.5–1.5Å), moderately for Si–Si pairs (1.5–2.0Å), and only marginally for O–O pairs. The degree of improvement in physicality directly reflects this nonuniform coverage. As shown in Figure 6(a), the finetuned MLIP achieves marked improvement over the baseline for Si–O and Si–Si interactions, particularly in the repulsive region where the baseline model extrapolates poorly. In contrast, improvement for O–O interactions is negligible, which we attribute to the limited coverage of O–O pairs below 1Å in the augmented data set. This limitation is intrinsic to our two-step perturbation algorithm: since O–O is rarely the closest pair in silica structures, the algorithm seldom generates configurations with compressed O–O distances, resulting in insufficient EAM knowledge transfer for this interaction. An analogous pattern is observed in MPTrj data sets (SI Section B), where similarly infrequent pairs such as Mn–Li also exhibit limited short-range coverage. Addressing this sampling asymmetry for minority pairs remains an avenue for future improvement.

## 4 Discussion

### 4.1 Computational Overhead

A key advantage of EAM-based pretraining is avoiding computationally expensive DFT labeling of augmented data sets. Although EAM training and pretraining introduce overhead, EAM’s efficiency compared to DFT makes the approach computationally advantageous. Using the MPTrj Subset with TorchMD as an example, we provide timing estimates: training EAM for 200 epochs requires 713 s (12 min), augmenting data 25× takes 73 s, and labeling 11,775 structures with EAM requires 234 s (4 min). Subsequent TorchMD pretraining on augmented data takes 16 h (50 epochs), and finetuning on the original MPTrj data set (10%) requires 2.4 h.

Note that the 16-h pretraining overhead applies regardless of labeling source (EAM or DFT). However, DFT labeling of 11,775 structures (11–104 atoms) would require substantially more resources. Our total overhead (EAM training + augmentation + EAM labeling + pretraining) is approximately 17 h versus 2.4 h for baseline training—a practical cost that avoids prohibitive DFT expenses. All measurements used a single NVIDIA A100 40GB GPU.

### 4.2 Limitations and Future Directions

While our physics-informed pretraining demonstrates clear advantages on the selected data sets, several limitations merit discussion. The method’s effectiveness depends critically on the accuracy and physical robustness of the labeling model. EAM performs poorly for high elemental complexity; we therefore limited evaluation to data sets with four or fewer unique elements. When EAM energy and force errors are significant, pretraining benefits may diminish.

A separate line of work develops foundational MLIPs trained on vast computational data sets, including SevenNet,^22^ MatterSim,^25^ ORB,^26^ EquiformerV2,^23^ and MACE.^75^ We note that these models fall outside the physics-informed pretraining framework studied here: unlike EAM, U-MLIPs do not encode explicit physical constraints in their functional form, and any physical regularities they capture are learned implicitly from training data rather than imposed by construction. Whether such implicit knowledge can be transferred to a student MLIP to enhance stability remains an open question. Recent studies document potential energy surface (PES) softening, i.e., energy and force underprediction from biased near-equilibrium sampling, in current foundational models,^34^ and Focassio et al.^76^ show that while these models excel at bulk predictions, they struggle in out-of-distribution scenarios such as surface energy calculations. Investigating whether U-MLIPs can serve as effective teachers within a pretraining-as-knowledge-transfer pipeline is therefore a distinct research direction beyond the scope of this work.

A second important limitation concerns the scope of systems to which our method is applicable. Because our approach relies on EAM as the pretraining teacher, its applicability is bounded by the regimes in which an empirical potential can be reliably fit to the target chemistry. For bulk and condensed-phase systems with well-defined bonding environments, such as systems evaluated in this work, EAM provides a physically reasonable foundation that successfully transfers useful inductive biases to the MLIP. However, for systems where simple empirical potentials are unavailable, poorly parametrized, or fundamentally inadequate, including surface reactions, bond-breaking and bond-forming events in catalysis, charged or open-shell species, our pretraining framework cannot be expected to provide meaningful physical guidance, and may in some cases inject misleading priors. We therefore caution against interpreting our results as evidence of universal applicability. The contributions of this work are most appropriately framed as a demonstration that, when a reasonable empirical potential exists for the target system, leveraging it through pretraining is a computationally inexpensive and effective route to improving MLIP stability and reliability in MD. Extending this paradigm to chemistries beyond EAM’s reach will require either more expressive empirical or semiempirical teachers (e.g., reactive force fields, tight-binding methods) or, as discussed above, foundational MLIPs whose own out-of-distribution behavior is sufficiently characterized.

A third limitation concerns the coverage of very short-range (e.g., below 1Å) interatomic distances in the training data. As illustrated in Figure 6, the pretraining and fine-tuning data sets contain effectively no pair configurations with interatomic distances below approximately 1.5Å, which limits the finetuned model’s ability to learn physically correct short-range repulsion. This is visible, for example, in the O–O pair interaction curve, where the finetuned TorchMD-Net model exhibits a substantially weaker repulsive wall than the reference EAM potential. To investigate whether targeted data augmentation could address this, we injected compressed diatomic geometries with pair distances sampled uniformly from 0.5 to 1.5Å into the pretraining data set. While this modestly improved the shape of short-range pair interaction curves, it in several cases degraded the downstream MD stability metrics across NVT and NPT ensembles, and thus this simple fix does not work as intended. We attribute this to the difficulty of reconciling highly compressed, unphysical geometries with the bulk near-equilibrium distribution that dominates training. We therefore recommend that future work address this limitation more carefully, for instance by constructing a dedicated short-range pretraining stage or by using a repulsive correction that is fitted separately and applied as a prior, rather than relying on sparse augmentation of the main training set.

## 5 Conclusion

This work presents a physics-informed pretraining methodology leveraging EAM potentials to enhance MLIP robustness and stability. Our pretraining-finetuning pipeline trains MLIPs on EAM-labeled augmented data sets to acquire physical knowledge before finetuning on quantum mechanical reference data.

Comprehensive evaluation across three material systems and three MLIP architectures demonstrates consistent improvements in both prediction accuracy and MD trajectory stability compared to baseline models, SAM optimization, and direct EAM-MLIP hybridization. The method is particularly effective at reducing unphysical behaviors during MD simulations, substantially decreasing atom overlap incidents and Lindemann index violations across all systems. Visualization of potential energy landscapes and force vector analysis reveals that pretraining enables MLIPs to learn physics-consistent predictions aligned with EAM profiles, improving out-of-distribution performance.

While current implementation is limited by EAM accuracy constraints, this work establishes a viable framework for incorporating physics-based knowledge from low-fidelity data into MLIP training. The integration of classical physics knowledge through pretraining represents a promising direction for developing robust MLIPs in computational materials science. A natural broader question is thus whether the pretraining-as-knowledge-transfer pipeline can be extended to teachers that lack explicit physical structure, such as universal MLIPs. We emphasize that such an extension would no longer constitute physics-informed pretraining in the sense developed here, and its effectiveness cannot be assumed given recent evidence of unphysical behavior in foundational models under out-of-distribution conditions. Investigating this distinction is left to future work.

## 6 Data and Software Availability

Data and model checkpoints supporting the results presented in this study are available at https://huggingface.co/datasets/qzheng75/EAM-Physical-MLIP/tree/main. The CGCNN and TorchMD checkpoints can be used with the MatDeepLearn package,^77^ while the M3GNet checkpoints are provided for integration with the MatterTune package.^78^ The implementation of the EAM potential under the MatDeepLearn framework is available at https://github.com/Fung-Lab/MatDeepLearn_dev/blob/eam_stable_md/matdeeplearn/models/eam_interaction.py and a sample implementation of the two-step data augmentation process can be found at https://github.com/Fung-Lab/MatDeepLearn_dev/blob/eam_stable_md/scripts/augmentation.py.

## Supplementary Material



## Notes

Experimental configuration parameters (augmentation factors, perturbation strength ranges, number of atomic pairs and sites) for constructing pretraining data sets across silica, phosphorus, and MPTrj systems for all three MLIP architectures.Pairwise potential energy landscape comparisons for phosphorus and MPTrj data sets across M3GNet and TorchMD-Net architectures, showing the potential energy surface of the vanilla and the fine-tuned model relative to EAM reference curves.Pair distance distribution comparisons between original and augmented training sets for all element pairs across silica, MPTrj, and phosphorus data sets, including both full and downsampled versions.Trajectory stability and prediction accuracy benchmarks for a direct additive EAM-MLIP hybrid architecture, with force vector angular distribution analysis comparing force alignment between direct combination and pretraining-finetuning approaches.Data efficiency ablation study comparing pretrained TorchMD-Net models fine-tuned on 5–10% of DFT data against vanilla baselines trained on increasing data fractions, evaluated by MD trajectory stability criteria.Standalone EAM potential evaluation including energy, force, and stress prediction errors on DFT reference data and MD trajectory stability assessments across all data sets and data regimes, with failure mode analysis.Hyperparameter specifications for CGCNN, TorchMD-Net, and M3GNet architectures used across all data sets (PDF)
