# Supplementary material for: Improving Reliability of Machine Learning Interatomic Potentials with Physics-Informed Pretraining
Source: J Chem Inf Model. 2026 Aug 28;66(17):10503–16. doi: 10.1021/acs.jcim.6c00826 (PMC13580122; doi:10.1021/acs.jcim.6c00826)
Supplement: Supplementary file 1 [file ci6c00826_si_001.pdf]

Improving Reliability of Machine Learning Interatomic Potentials With Physics-Informed Pretraining

Supporting Information

Qianyu Zheng[†], **Victor Fung**[†*]
[†]College of Computing, Georgia Institute of Technology, Atlanta, GA 30332, USA
[†*]School of Computational Science and Engineering, Georgia Institute of Technology, GA 30332, USA

A Detailed experimental setup for constructing the pretraining dataset

The comprehensive experimental configurations are summarized in Table S1. The notation $[a, b)$ represents a linearly spaced sequence of perturbation strengths generated using numpy's linspace function, where a is the starting value, b is the endpoint (exclusive), and the number of points equals the augmentation factor n_{aug}. For instance, $[a, b)$ with five augmentations generates the sequence $\{1.0, 0.8, 0.6, 0.4, 0.2\}$ Å. Each augmentation round applies our dual-mode perturbation algorithm with the corresponding strength values, resulting in a pretraining dataset that is n_{aug} times larger than the original quantum mechanical reference data.

Dataset	MLIP	Usage	n aug	n pairs	Pull	n sites	Perturb
Silica	CGCNN	100%	5	4	[1.0, 0.0)	5	[1.0, 0.0)
	TorchMD	10%	5	4	[1.0, 0.0)	5	[1.0, 0.0)
	M3GNet	10%	5	2	[1.0, 0.0)	5	[1.0, 0.0)
Phosphorus	CGCNN	100%	5	2	[1.0, 0.0)	5	[1.0, 0.0)
	TorchMD	5%	20	2	[2.0, 0.0)	5	[2.0, 0.0)
	M3GNet	5%	5	2	[1.0, 0.0)	5	[0.5, 0.0)
MPtrj Subset	CGCNN	100%	10	2	[0.5, 0.0)	5	[0.25, 0.0)
	TorchMD	10%	25	2	[1.0, 0.0)	5	[0.5, 0.0)
	M3GNet	10%	25	2	[1.0, 0.0)	5	[0.5, 0.0)

Table S1: Experimental configuration parameters for physics-informed pretraining. Usage indicates the fraction of original dataset used for training; n aug is the number of augmentation rounds applied; n pairs and n sites are the number of close atomic pairs identified for pulling and the number of sites for random perturbation, respectively; Pull and Perturb specify the strength ranges for atomic pulling and random perturbation, respectively.

B Energy landscape validation across datasets and architectures

To complement the silica (SiO_2) energy curves shown in Figure 6 of the main text, we provide here the corresponding pairwise potential energy landscapes for the phosphorus (P) and MPTrj (Li, Mn, O, P) datasets. To demonstrate that the observed improvements in physicality are not specific to a single model family, we report results for two architectures: M3GNet, an invariant graph neural network, and TorchMD-Net, an equivariant transformer. In each panel, the solid blue curve corresponds to the vanilla model and the dashed orange curve corresponds to the model after fine-tuning with our procedure.

M3GNet Potential Energy Surfaces **MPTrj (Li, Mn, O, P; top two rows):** The vanilla model already exhibits a qualitatively repulsive short-range wall for all ten unique pair types, but with a pronounced oscillatory spike near ∼1 Å that is absent from the EAM reference. Fine-tuning consistently eliminates this artifact and raises the repulsive wall to magnitudes in closer agreement with the EAM, with clear improvement across all pairs including homoatomic ones (Li-Li, Mn-Mn, O-O, P-P) and metal-metal pairs (Li-Mn). **Phosphorus and silica (bottom row):** The fine-tuned model recovers strongly divergent repulsion for O-O, O-Si, and Si-Si pairs, in close agreement with the EAM reference and consistent with Figure 6 of the main text. The P-P channel shows partial recovery but retains a residual bump near ∼1.5 Å, consistent with

*Corresponding author: victorfung@gatech.edu

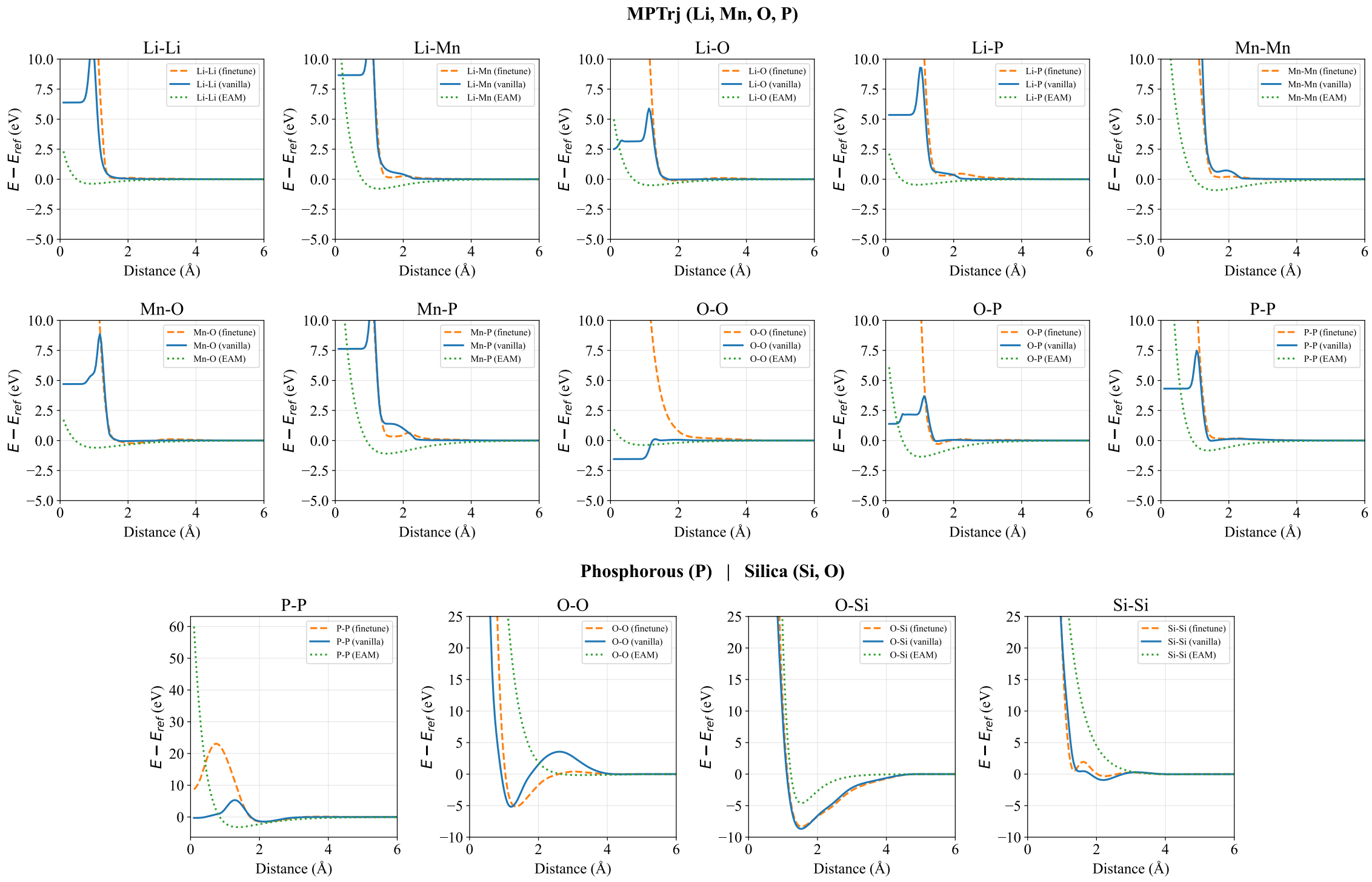

Figure S1: Pairwise potential energy curves from the M3GNet model, comparing the vanilla model (solid blue), the fine-tuned model (dashed orange), and the EAM potential (dotted green) across all benchmark datasets. We follow Figure 6 in the main text to plot the energy with respect with the reference energy $(E - E_{ref})$.

Figure S5 that homoatomic phosphorus pairs remain underrepresented in the very short-range regime of the pretraining dataset.

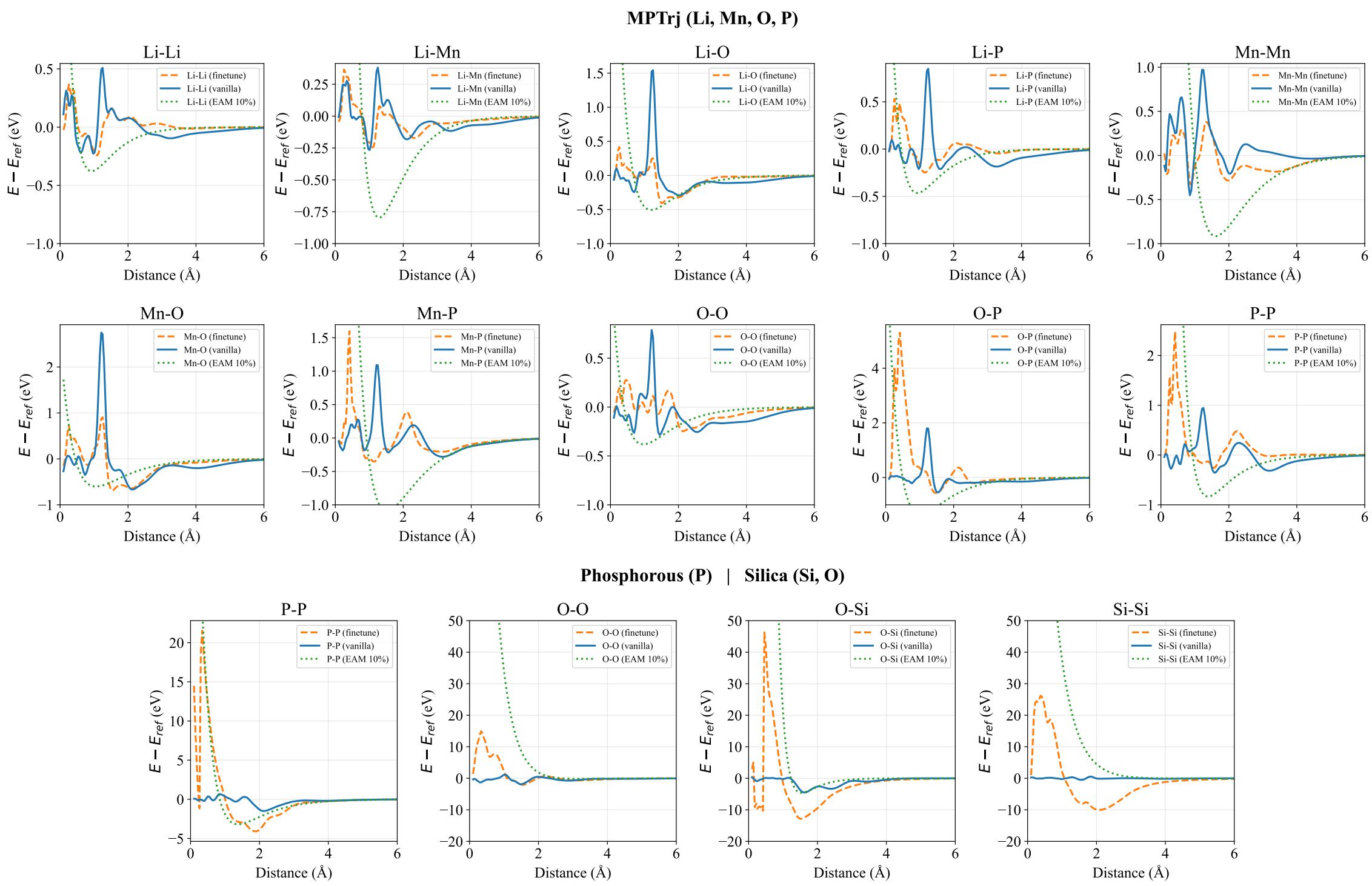

Figure S2: Pairwise potential energy curves from the TorchMD-Net model, comparing the vanilla model (solid blue), the fine-tuned model (dashed orange), and the EAM potential (dotted green) across all benchmark datasets. We follow Figure 6 in the main text to plot the energy with respect with the reference energy $(E - E_{ref})$.

TorchMD-Net Potential Energy Surfaces **MPTrj (Li, Mn, O, P; top two rows):** The vanilla curves span only a few eV and exhibit spurious oscillatory features near typical bonding distances (∼1–2 Å) rather than a physically correct repulsive wall. Fine-tuning recovers smooth repulsive behavior for heterogeneous pairs involving at least one electronegative species, most notably Li-O, Li-P, Mn-P, O-P, P-P and Li-Mn, which are well-represented in the pretraining perturbation scheme. In contrast, some homoatomic and metal-metal pairs (Li-Li, Li-Mn, Mn-Mn) and the O-O pair show limited improvement, as these combinations are rarely the closest pair in MPTrj structures and thus receive insufficient short-range coverage during pretraining — an analog of the O-O limitation discussed for silica in Figure 6. **Phosphorus and silica (bottom row):** The fine-tuned model recovers the expected divergent repulsion at short distances for P-P, O-Si, and Si-Si pairs, in agreement with M3GNet results and with Figure 6 of the main text. The O-O pair again shows comparatively limited improvement, consistent with its under-representation in compressed configurations across all datasets.

Across both architectures and all three datasets, the fine-tuned models exhibit the same qualitative improvements highlighted for silica. The magnitude of the corrections at low interatomic distances varies with dataset chemical complexity and model architecture and within each dataset, but the trend is consistent in most interaction types. Together, Figures S1 and S2 confirm that the conclusions drawn from the silica case different model architectures and datasets.

C SHORT-RANGE COVERAGE OF THE AUGMENTED PRETRAINING DATASETS

To make the pretraining dataset exploration done in Figure 6(b) complete for all datasets (both full and downsampled versions), we present Figures S3a–S5 that compare, for every element pair, the distribution of its closest-contact (minimum) distance per structure in the original training set against that of the corresponding augmented set. In each panel the original distribution (blue) is sharply peaked near the equilibrium bond length, while the augmented distribution (orange) develops a broad shoulder extending toward shorter distances; the leftward extent of this shoulder is the new short-range coverage introduced by augmentation.

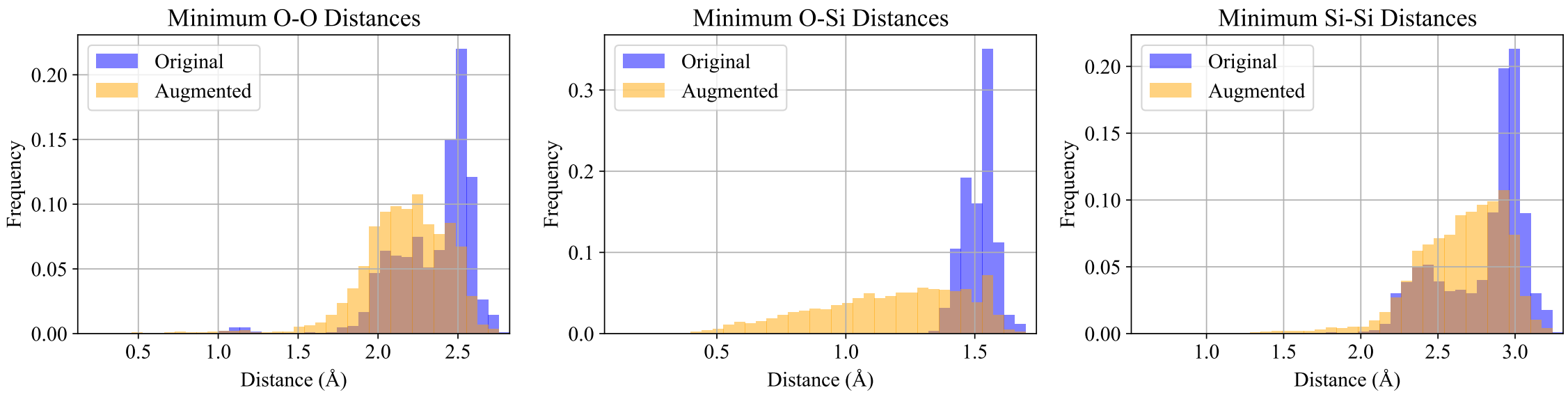

(a) Distribution of the closest-contact (minimum) interatomic distance per structure for the full silica (SiO_2) dataset

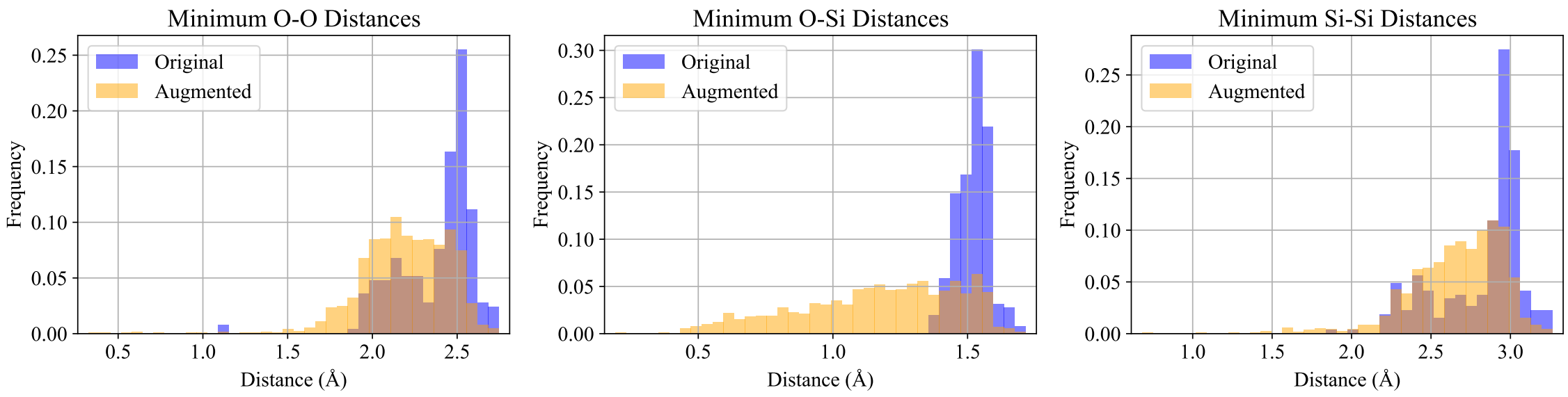

(b) Distribution of the closest-contact (minimum) interatomic distance per structure for the 10% downsampled silica (SiO_2) dataset

Figure S3: Closest-contact (minimum) distance analysis for the Silica dataset

A consistent picture emerges across the three chemical systems. Pairs involving the most electronegative species, which typically form the shortest bonds and are thus most often the closest pair in a structure, show the strongest leftward shift, whereas homo-atomic and metal–metal pairs, which are rarely the closest contact, shift comparatively little. This coverage asymmetry is the structural origin of the pair-by-pair differences in the fine-tuned energy landscapes of Figures S1 and S2: pairs that are well-augmented here are exactly those for which fine-tuning recovers a physically divergent repulsive wall, while poorly-augmented pairs show the most residual artifacts.

For **silica** (Figure S3a and S3b), the O–Si pair shows by far the strongest leftward shift, its augmented shoulder reaching well below the original peak, because O–Si is almost always the closest contact in a

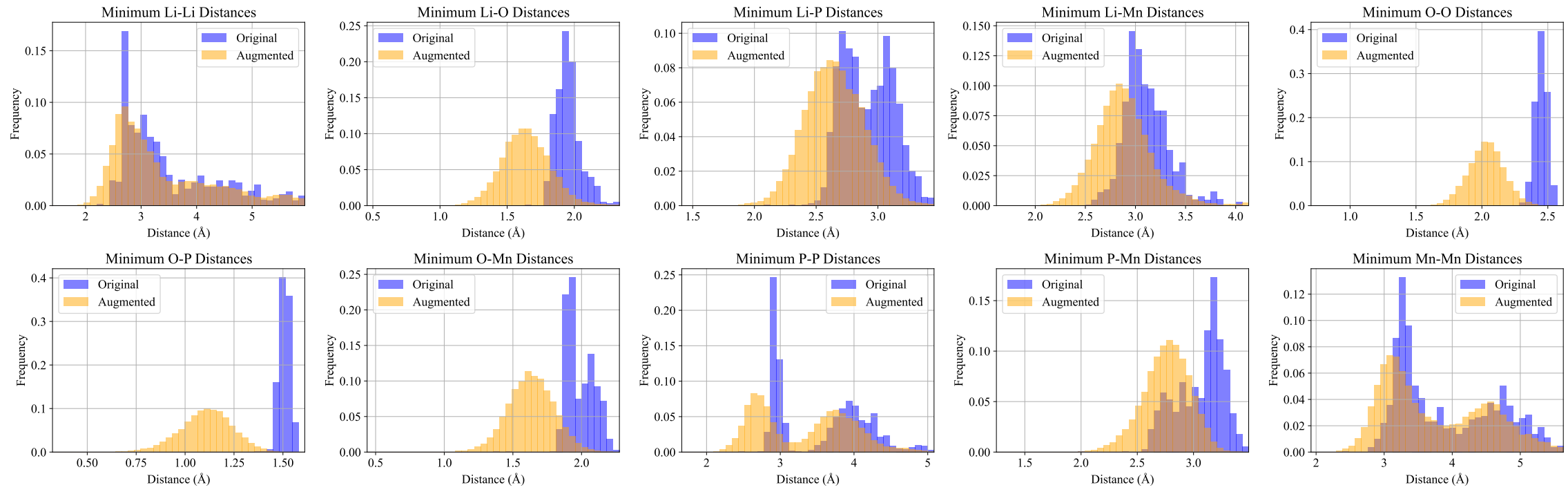

(a) Distribution of the closest-contact (minimum) interatomic distance per structure for the MPTrj subset dataset

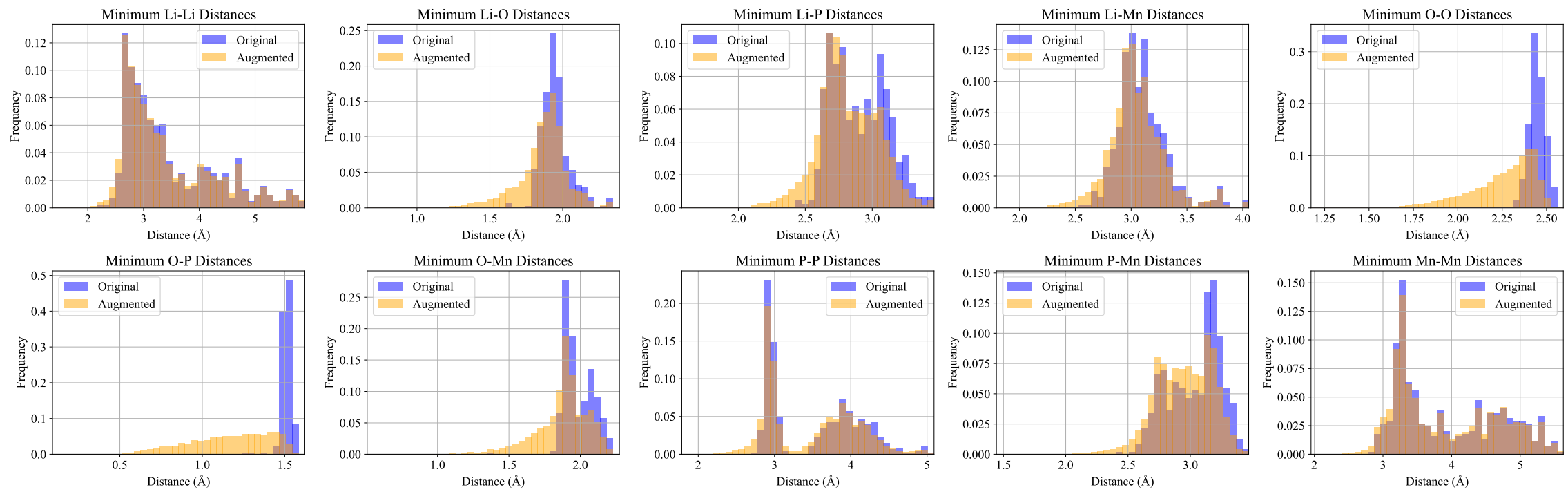

(b) Distribution of the closest-contact (minimum) interatomic distance per structure for the 10% downsampled MPTrj subset dataset

Figure S4: Closest-contact (minimum) distance analysis for the MPTrj subset dataset

silica structure. The Si–Si and O–O pairs are pulled shorter much less, consistent with the more significant unphysical O–O/Si–Si artifacts noted for silica in the main text. For the **MPtrj subset** (Figure S4a and S4b), the oxygen-bearing heteroatomic pairs — O–P, O–O, and O–Mn — exhibit the most significant short-range shift, while the metal–metal and homoatomic pairs (Li–Li, P–Mn, Mn–Mn) shift least. This is the direct dataset-level explanation for the TorchMD-Net observation in Section B that Li–Li and Mn–Mn benefit least from pretraining. The single-species **phosphorus** dataset (Figure S5) has only the P–P channel, whose augmented distribution is clearly pulled toward shorter contacts in both the full and 5% versions, in line with the strong P–P repulsion recovered after fine-tuning. In every case the data-constrained versions reproduce the same qualitative shift as their full counterparts, confirming that the augmentation injects short-range information in a chemically consistent manner even in the low-data regime.

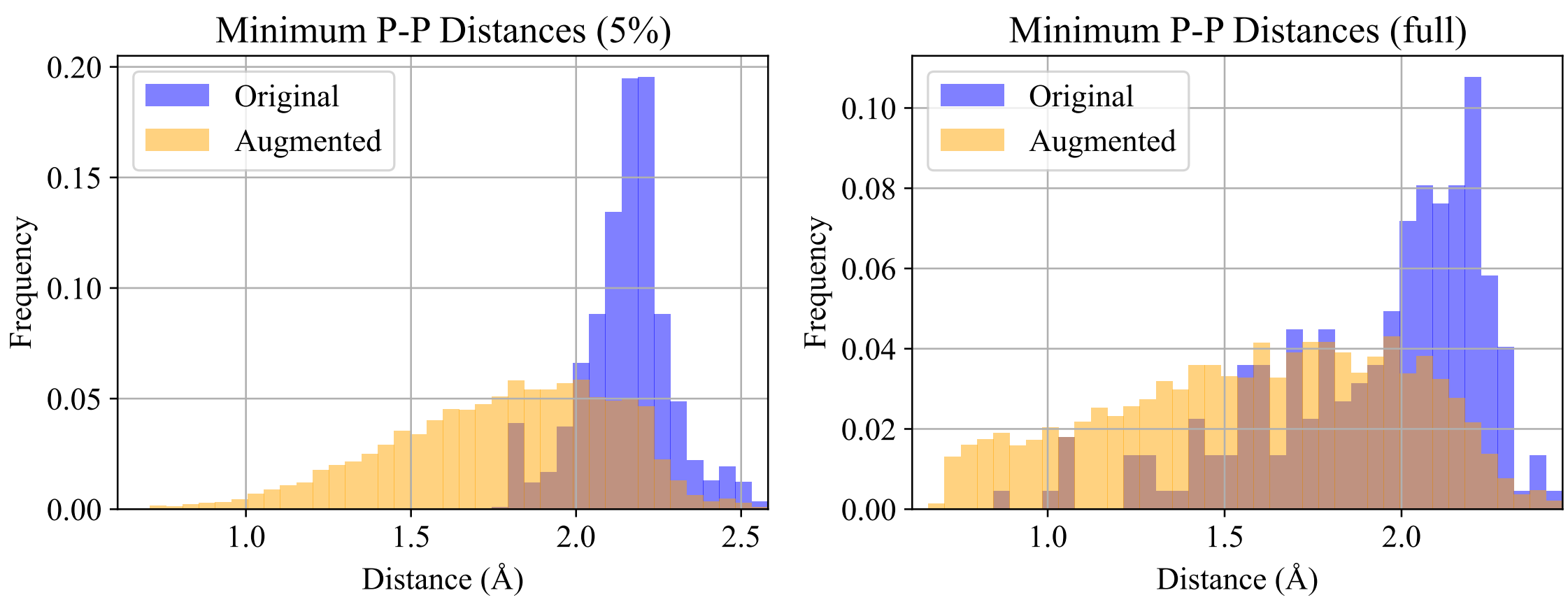

Figure S5: Distribution of the closest-contact (minimum) interatomic distance per structure for both the full and the 5% downsampled phosphorous dataset

Dataset	MD Setting	Setup	Atom Overlap	Lindemann Index
Silica (full)	NVT (2000K, 2fs)	CGCNN + EAM	3	1
	NPT (2000K, 2fs)		6	3
Silica (10%)	NVT (2000K, 2fs)	TorchMD + EAM	2	3
	NPT (2000K, 2fs)		9	8
Phosphorous (full)	NVT (1250K, 1fs)	CGCNN + EAM	39	67
Phosphorous (5%)	NVT (1250K, 1fs)	TorchMD + EAM	11	79
MPtrj Subset (full)	NVT (500K, 1fs)	CGCNN + EAM	100	0
	NPT (250K, 1fs)		47	47
MPtrj Subset (10%)	NVT (1000K, 1fs)	TorchMD + EAM	88	100
	NPT (750K, 1fs)		81	100

Table S2: Trajectory stability assessment for hybrid MLIPs across diverse datasets and simulation conditions. All molecular dynamics simulations were conducted for 5000 timesteps on 100 randomly sampled structures from each dataset's test set. Values represent the number of trajectories (out of 100) that exhibit unphysical behavior as detected by atom overlap and Lindemann index criteria. NVT and NPT simulations are performed with Langevin and Berendsen dynamics, respectively, as introduced in section 2.5.1, and implemented with the Atomic Simulation Environment (ASE) package.

D COMPARISON WITH DIRECT EAM INTEGRATION

At the early stage of our research, we investigated a seemingly easier approach to introduce chemical knowledge from traditional interatomic potentials by directly incorporating EAM-predicted energies as an additive term to the MLIP output, following the regularization strategy proposed in [1]. In this hybrid architecture, we first train the EAM potential independently, then jointly optimize both the EAM parameters and MLIP weights on quantum mechanical reference data. The total energy prediction combines contributions from both the MLIP and EAM components: $E_{total} = E_{MLIP} + E_{EAM}$. However, our evaluation across multiple datasets and MD simulation demonstrates that this hybrid approach yields inferior performance compared to our proposed sequential pretraining-finetuning methodology, as evidenced by both prediction accuracy metrics (Table S3) and trajectory stability assessments (Table S2).

Dataset	Setup	E Error	F Error	S Error	Total Error
Silica	CGCNN + EAM	2.970	0.193	0.00134	**9.754**
Silica (10%)	TorchMD + EAM	2.490	0.116	0.00111	5.875
Phosphorous	CGCNN + EAM	8.480	0.264	/	13.280
Phosphorous (5%)	TorchMD + EAM	8.480	0.208	/	13.280
MPtrj Subset	CGCNN + EAM	1.110	0.0901	0.00188	4.611
MPtrj Subset (10%)	TorchMD + EAM	4.540	0.0875	0.00188	**4.512**

Table S3: Prediction accuracy comparison for hybrid MLIPs combining MLIPs with EAM potentials. Energy errors are reported as mean absolute error (MAE) in eV, force errors in eV/Å, and stress errors in GPa. The total error represents a weighted combination of energy, force, and stress MAEs using weights 0.01:50:50, respectively. Dataset percentages indicate the fraction of available training data used for model development. The highlighted "Total Error" entries are experimental setups in which the direct-combination approach yields a lower total EF(S) prediction error compared to the pretraining-finetuning approach introduced in the main text.

The comprehensive evaluation across three diverse datasets and multiple simulation conditions demonstrates the clear superiority of our proposed physics-informed pretraining methodology over the direct EAM-MLIP combination approach. The trajectory stability assessment (Table S2) reveals the fundamental limitations of direct combination methods: the hybrid methods consistently exhibit substantially higher rates of trajectory instability across most tested systems compared to the proposed pretraining approach. These results demonstrate that sequential pretraining with EAM-derived knowledge provides superior physics-informed initialization that enhances both prediction accuracy and long-term simulation stability, whereas direct combination approaches fail to effectively leverage empirical potential knowledge and may harm trajectory physicality.

To explore the inferior performance of the direct combination approach, we investigated how the MLIP aligns with the EAM potential in the direct combination method and the proposed pretraining-finetuning

method. The angular distribution analysis reveals a fundamental incompatibility between direct EAM-MLIP combination approaches and highlights the effectiveness of our proposed pretraining methodology in achieving coherent physics-informed learning. For the direct combination approach, we performed inference with the hybrid model on test structures from each dataset and decomposed the total force predictions to separately extract the EAM and MLIP force contributions for each atom, then computed the angles between these concurrent force vectors using the dot product $\theta = \arccos\left(\frac{\mathbf{F}_{EAM} \cdot \mathbf{F}_{MLIP}}{|\mathbf{F}_{EAM}||\mathbf{F}_{MLIP}|}\right)$. The results, as shown in Figure S6, demonstrate that these two components frequently "fight against" each other, producing conflicting force directions that manifest as broad angular distributions with substantial density at large angles ($90 - 180°$), particularly evident in the Silica and MPtrj datasets where direct combination methods exhibit significant force misalignment. This may explain the especially inferior MD stability performance of the hybrid MLIP in these two datasets. In contrast, for our pretraining approach, we computed angles between force predictions from the finetuned MLIP and the EAM potential used for pretraining dataset generation when both models independently performed inference on the test structures. The angular distributions are more concentrated near small angles, which indicates a more consistent force alignment across all tested systems and demonstrates that our "soft knowledge injection" strategy through sequential pretraining enables the MLIP to internalize and harmonize with EAM physics principles, rather than competing against them. The pretraining methodology thus facilitates smoother EAM integration by allowing the MLIP to learn representations that respect rather than contradict the underlying empirical potential, resulting in more stable and physically consistent predictions during inference.

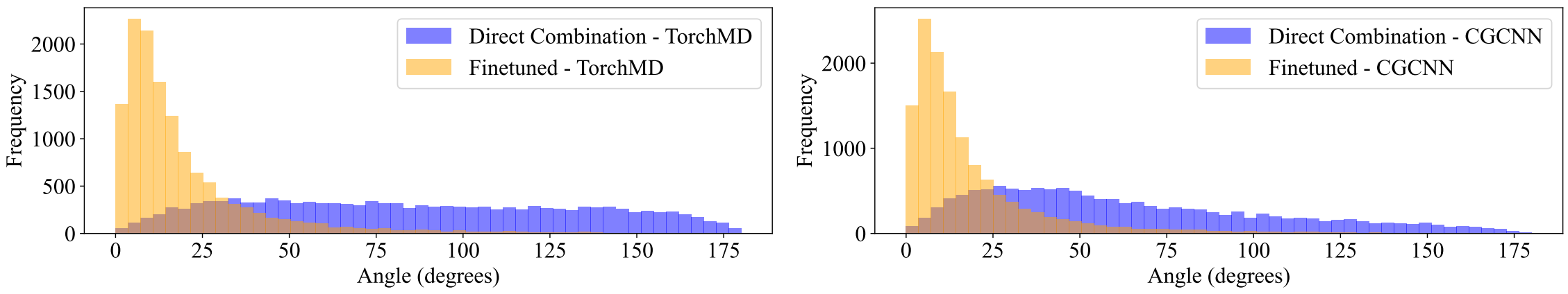

(a) Distribution of EAM-MLIP Predicted Forces Angles in Silica Dataset

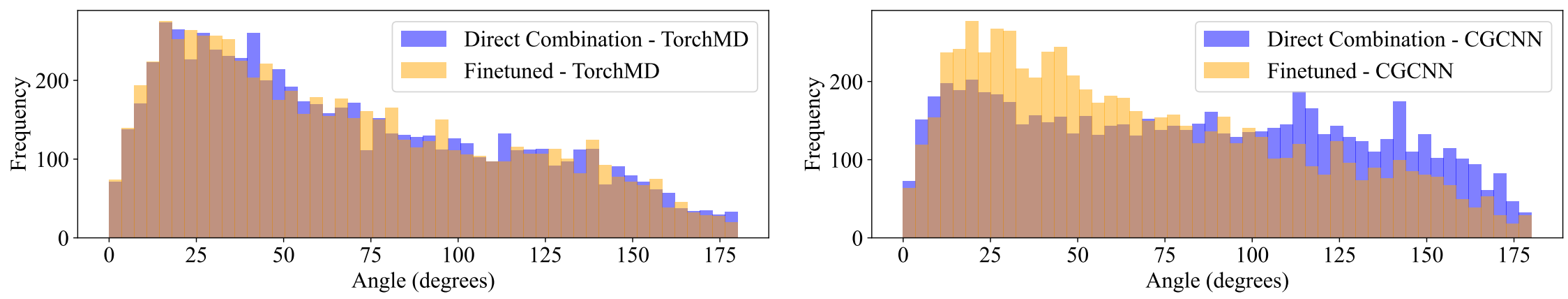

(b) Distribution of EAM-MLIP Predicted Forces Angles in Phosphorous Dataset

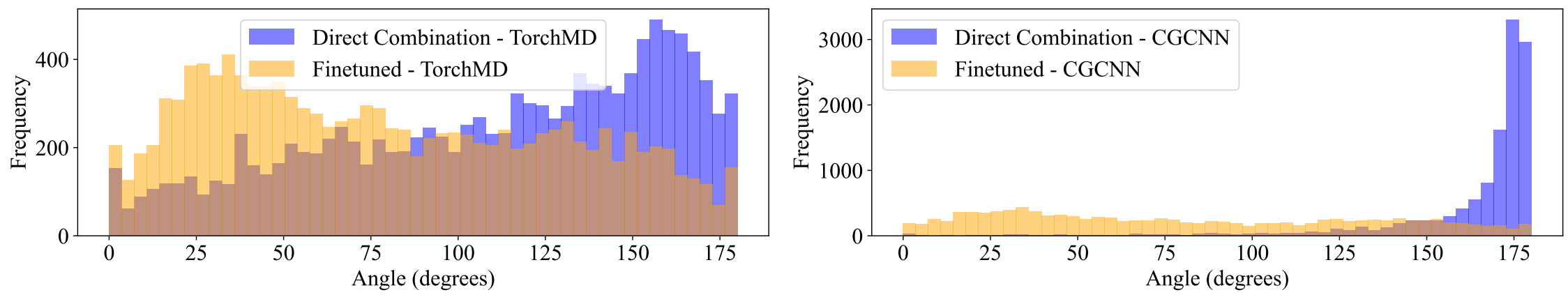

(c) Distribution of EAM-MLIP Predicted Forces Angles in MPtrj Subset Dataset

Figure S6: Angular distribution analysis of force predictions comparing direct EAM combination versus pretraining approaches across three datasets. The histograms show the distribution of angles between MLIP-predicted and EAM-predicted force vectors for individual atoms, where $0°$ indicates perfect alignment and $180°$ represents complete opposition. Orange distributions represent models trained with our proposed pretraining-finetuning methodology, while blue distributions show results from direct EAM-MLIP combination approaches.

E Data Efficiency of the EAM Pretraining Method

A central motivation for our pretraining approach is the resource-constrained regime in which large, high-coverage DFT datasets are not available and cannot feasibly be generated. This setting arises frequently in practice, for example when developing MLIPs for newly studied material systems, custom chemistries, or DFT settings that differ from those used in existing public datasets. In such cases, the bottleneck is precisely the cost of generating additional DFT labels, and strategies that rely on large-scale data coverage are not directly applicable. Our method is therefore designed to be complementary to large-scale data scaling rather than to compete with it: the two strategies address different points on the data-availability spectrum.

To quantify the data-efficiency advantage of our approach in this regime, we conducted a controlled ablation on the phosphorus and the MPTrj datasets using TorchMD-NET. We vary the fraction of DFT training data used by the vanilla baseline while keeping our pretrained model fixed at only 5% and 10% of the DFT data. Stability is reported as the number of trajectories (out of 100) violating the atom-overlap and Lindemann-index criteria under the same simulation protocol described in Section 3. Table S4 reports results on the phosphorus dataset and the MPTrj subset dataset.

Table S4: Stability ablation on the phosphorus and the MPTrj dataset under NVT simulation. We compare vanilla TorchMD-NET trained on increasing fractions of DFT data against our pretrained model fine-tuned on the undersampled datasets. Overlap and Lindemann columns report the number of trajectories (out of 100) violating the respective stability criteria; lower is better. The pretrained models with 5% data of the phosphorous dataset and 10% of the MPTrj dataset match the performance of the vanilla baseline trained on 100% of the data.

Setting	Setup	DFT data used	Overlap	Lindemann
Phosphorus (NVT)	Vanilla TorchMD	5%	9	42
	Vanilla TorchMD	25%	16	27
	Vanilla TorchMD	50%	17	36
	Vanilla TorchMD	100%	6	24
	**TorchMD + pretraining**	**5%**	**6**	**24**
MPTrj (NVT)	Vanilla TorchMD	10%	70	98
	Vanilla TorchMD	25%	100	100
	Vanilla TorchMD	50%	38	97
	Vanilla TorchMD	100%	19	**39**
	**TorchMD + pretraining**	**10%**	**11**	40

Across both datasets, the pretrained model trained on only 5% or 10% of the DFT data matches the stability of the vanilla model trained on the *full* dataset, and outperforms vanilla baselines trained on 25% and 50% of the data. Achieving equivalent stability through data scaling alone therefore requires roughly $10\times$ to $20\times$ more DFT labels than our pretraining approach. This is precisely the regime in which simple data scaling is least applicable: when the cost-limiting step is generating DFT labels for a specific system, a method that delivers the same stability with a fraction of the DFT budget offers a more direct and effective route to robust MLIPs.

F EAM POTENTIAL AS A STANDALONE POTENTIAL

In this section, we evaluate the capabilities of the EAM potential as a standalone potential. We first report the prediction errors of the trained EAM potentials on the DFT reference data for each dataset. Table S5 summarizes the energy, force, and stress mean absolute errors (MAEs) obtained after fitting EAM to each dataset, evaluated under both the full-data and reduced-data regimes used in the main text.

As expected, the EAM potential exhibits substantially higher errors than the MLIPs reported in Table 2 of the main text, which reflects the limited expressivity of its fixed functional form. Energy errors are particularly large for the Phosphorous and reduced-data Silica/MPtrj cases, where the diversity of local environments exceeds what the compact EAM parameterization can capture quantitatively. Nevertheless, as demonstrated throughout the main text, the EAM potential's role in our pipeline is not to provide quantitatively accurate labels but to supply physically reasonable energy landscapes, particularly in repulsive regions underrepresented in DFT training data, that bias the MLIP toward physically consistent solutions during pretraining.

We then evaluate the capability of the EAM potential to empower MD simulations. With the same configurations as described in the main text adopted, we conduct experiments on all three datasets, both the full and the undersampled version and present the results in Table S6.

The instability of the EAM potential in MPTrj subset MD simulations stems not from a failure of short-range repulsion but from gradual thermal melting and pressure-control failures. To verify this, we analyzed every failed trajectory frame by frame, tracking the time to atomic overlap, the Lindemann index, and simulation box stability. For each trajectory, we measured the transition time from a clearly intact structure (closest atomic pair at least 90% of its initial spacing) to an overlapping one (spacing below 50% of initial). Across all overlap events, this transition spans tens to several hundred MD steps (ranging from $\sim$78 steps in the mildest setting to $\sim$710 in the most failure-prone), with no event completing in three steps or fewer. This rules out a weak repulsive wall as the cause. The NPT failures constitute a second independent failure mode, where pressure-control amplifies volume fluctuations that EAM cannot adequately damp, causing

Table S5: Prediction accuracy of the trained EAM potentials on DFT reference data for each dataset. Energy errors are reported in eV, force errors in eV/Å, and stress errors in GPa. Total error is a weighted combination using weights 0.01:50:50 for energy:force:stress (0.01:50 for the Phosphorous dataset due to missing stress labels). Dataset percentages indicate the fraction of training data used for EAM fitting, matching the regimes used in the main text.

Dataset	Usage	E Error	F Error	S Error	Total Error
Silica	100%	9.700	0.461	0.00872	23.588
	10%	11.300	0.466	0.00713	23.786
Phosphorous	100%	13.500	0.433	/	21.792
	5%	48.500	0.670	/	33.991
MPtrj Subset	100%	2.610	0.0975	0.00281	5.043
	10%	71.700	0.0977	0.00413	5.897

**Dataset**	**MD Setting**	**Atom Overlap**	**Lindemann Index**
Silica (full)	NVT (2000K, 2fs)	2	3
	NPT (2000K, 2fs)	16	16
Silica (10%)	NVT (2000K, 2fs)	4	4
	NPT (2000K, 2fs)	22	21
Phosphorous (full)	NVT (1250K, 1fs)	33	71
Phosphorous (5%)	NVT (1250K, 1fs)	10	60
MPtrj Subset (full)	NVT (500K, 1fs)	18	90
	NPT (250K, 1fs)	100	100
MPtrj Subset (10%)	NVT (1000K, 1fs)	97	99
	NPT (750K, 1fs)	96	96

Table S6: Trajectory stability assessment for EAM as a standalone potential across the three datasets. All molecular dynamics simulations were conducted for 5000 timesteps on 100 randomly sampled structures from each dataset's test set. Values represent the number of trajectories (out of 100) that exhibit unphysical behavior as detected by atom overlap and Lindemann index criteria. NVT and NPT simulations are performed with Langevin and Berendsen dynamics, respectively, as introduced in section 2.5.1, and implemented with the Atomic Simulation Environment (ASE) package.

collapse of the lattice. Neither failure mode reflects a deficiency in EAM's repulsive wall; both reflect the limitations of EAM's functional form when applied outside its original design domain of close-packed metallic systems.

As a conclusion, the EAM potential is itself not a fully stable simulator on our datasets, as pure-EAM MD produces non-trivial atom overlaps and Lindemann violations on all three systems, with the most severe failures on the multi-element MPtrj subset where EAM's functional form is least adequate. Second, EAM's standalone EFS errors are roughly an order of magnitude larger than those of the MLIPs (Table 2). A hybrid model described in Section D therefore couples the MLIP to a teacher that is neither quantitatively accurate nor independently stable on these systems, which leads to inferior performance compared to the pretraining approach described in the main text. In this work, EAM should be best understood as a teacher of physical structure, not as a stable simulator, and pretraining is the mechanism that separates the transferable physical knowledge from EAM's quantitative limitations.

G Hyperparameters for MLIP architectures

In this section, we presented detailed hyperparameters for the selected MLIP architectures. The configurations for each architecture are transferrable to all three datasets.

CGCNN: The CGCNN architecture employs a 4-layer graph convolutional network with hidden dimensions of 100 in the convolutional layers, followed by a post-GNN MLP with hidden dimension of 150. The model includes 1 pre-fully connected layer and 3 post-fully connected layers and utilizes global addition pooling applied late in the architecture with SiLU activation functions.

TorchMD: The TorchMD architecture employs an 8-layer graph neural network with 128 hidden channels and 50 radial basis functions, incorporating 8 attention heads with both SiLU activation and attention activation functions. The model includes 2 post-processing layers with 128 post-hidden channels and utilizes global addition pooling applied late in the architecture with additive aggregation.

M3GNet: The selected M3GNet architecture has the same configuration as the backbone model of the foundational model MatterSim-v1.0.0-1M. Details about the architecture can be found at [2].

REFERENCES

[1] Fu, W. *et al.* Enhancing molecular energy predictions with physically constrained modifications to the neural network potential. *J. Chem. Theory Comput.* **20**, 4533–4544 (2024).

[2] Yang, H. *et al.* Mattersim: A deep learning atomistic model across elements, temperatures and pressures. *arXiv preprint arXiv:2405.04967* (2024). URL `https://arxiv.org/abs/2405.04967`. `2405.04967`.
